# Cell wall proteome analysis of *Mycobacterium smegmatis *strain MC2 155

**DOI:** 10.1186/1471-2180-10-121

**Published:** 2010-04-22

**Authors:** Zhiguo He, Jeroen De Buck

**Affiliations:** 1 Department of Production Animal Health, Faculty of Veterinary Medicine, University of Calgary, 3330 Hospital Drive NW, Calgary, AB T2N 4N1, Canada

## Abstract

**Background:**

The usually non-pathogenic soil bacterium *Mycobacterium smegmatis *is commonly used as a model mycobacterial organism because it is fast growing and shares many features with pathogenic mycobacteria. Proteomic studies of *M. smegmatis *can shed light on mechanisms of mycobacterial growth, complex lipid metabolism, interactions with the bacterial environment and provide a tractable system for antimycobacterial drug development. The cell wall proteins are particularly interesting in this respect. The aim of this study was to construct a reference protein map for these proteins in *M. smegmatis*.

**Results:**

A proteomic analysis approach, based on one dimensional polyacrylamide gel electrophoresis and LC-MS/MS, was used to identify and characterize the cell wall associated proteins of *M. smegmatis*. An enzymatic cell surface shaving method was used to determine the surface-exposed proteins. As a result, a total of 390 cell wall proteins and 63 surface-exposed proteins were identified. Further analysis of the 390 cell wall proteins provided the theoretical molecular mass and p*I *distributions and determined that 26 proteins are shared with the surface-exposed proteome. Detailed information about functional classification, signal peptides and number of transmembrane domains are given next to discussing the identified transcriptional regulators, transport proteins and the proteins involved in lipid metabolism and cell division.

**Conclusion:**

In short, a comprehensive profile of the *M. smegmatis *cell wall subproteome is reported. The current research may help the identification of some valuable vaccine and drug target candidates and provide foundation for the future design of preventive, diagnostic, and therapeutic strategies against mycobacterial diseases.

## Background

Although *Mycobacterium smegmatis *was originally isolated from humans, this fast-growing mycobacterium species is mostly nonpathogenic and has been used as a model to investigate mycobacterial physiology [1,2]. This fast-growing nonpathogenic bacterium is particularly useful in studying basic cellular processes of relevance to pathogenic mycobacteria, such as *Mycobacterium tuberculosis*, *M. avium subsp. paratuberculosis *and *M. leprae*, respectively the causative agent of tuberculosis, Johne's disease and leprosy. Although the genome sequencing of *M. smegmatis *is completed, much is unknown about the mechanisms controlling growth in mycobacterial species. As occurs with all free living bacteria, cells of *M. smegmatis *are surrounded by a cell wall responsible for providing their shape. The wall also provides protection to the cell to withstand the difference in osmotic pressure with the medium, and against other physical and chemical aggressions. Nevertheless, the cell wall must not be considered as a static structure; its chemical composition and the assembly of the different macromolecules that make it up are modified during cell growth and morphogenesis. A characteristic feature of mycobacteria is the thick, waxy cell wall, a highly impermeable outer surface, which enables mycobacteria to survive in extreme environmental conditions and the presence of antibiotics. The cell envelope structure of Mycobacteria is different from other gram positive bacteria, by the fact that it has two lipid layers, one being a regular inner membrane, the second being a layer mainly consisting of mycolic acids. This mycomembrane is very tightly connected to the peptidoglycan and arabinomannan inner layers of the cell wall. The surface is very complex, composed of proteins, sugars, and lipids that are in part conserved across the Mycobacterial genus. While many of the cell wall proteins are burried inside the cell wall, some are surface exposed and likely play an even greater role in many vital processes such as cell-cell interactions, ion and nutrient transport and cell signaling, and participate in the key pathogenically relevant cellular mechanisms. Many proteins required for the pathogenicity of Mycobacteria are surface proteins that are involved in lipid metabolism and transport across the cell envelope [3,4]. Surface proteins are exposed to the external environment. As a result, these proteins are ideally positioned to protect the bacterium or to modify the host immune response to the bacillus. So research on the cell wall proteome of *M. smegmatis *provides promising candidates for vaccine and drug development against pathogenic *Mycobacterium spp*., especially since it turns out that bacterial cell envelope together with plasma membrane proteins constitute the majority of currently known drug targets [5,6].

While other studies have used 2 dimensional liquid chromatography to increase the number of protein identifications in a complex mixture by tandem mass spectrometry [7,8], we have chosen for a proteomic shotgun approach where SDS-PAGE precedes LC-MS/MS to resolve the *M. smegmatis *cell wall proteome. Other studies have previously used this approach to resolve mycobacterial membrane proteins [9-12]. The goal of this study was to improve the identification of mybacterial cell wall and cell wall-associated proteins in Mycobacteria by analyzing the model organism *Mycobacterium smegmatis*.

## Results & discussion

### High-throughput identification of cell wall proteins with SDS-PAGE + LC-MS/MS

Traditionally, proteomic analyses of cell wall samples involve the resolution of proteins using 2-DE followed by the identification of resolved proteins by MS [13]. However, a big proportion of cell wall proteins are membrane bound, and it is generally agreed that membrane proteins are highly underrepresented in 2 dimensional electrophoresis (2-DE) [14]. In view of the poor performance of the 2-DE technique for membrane proteins and because the electrophoretic resolution of 2-DE by contaminating mycolates and other cell wall components [15], an alternative approach for the analysis of the cell wall proteome, shotgun LC-MS/MS method, was conducted. Cell wall proteins were first separated by SDS-PAGE according to their molecular weight followed by in-gel digested with trypsin into complex peptide mixture, and then the mixture was analyzed directly by LC-MS/MS. Subsequently, protein identifications were determined by database searching software [16]. Our experiments led to the identification of a much wider range of proteins in cell wall fraction than those identified using the conventional 2-DE based method and can therefore be used as a comprehensive reference for *Mycobacterium *spp. cell wall proteomic studies. To avoid false-positive hits, we applied strict criteria for peptide and proteins identification. Additional file 1 shows the identified proteins in detail. In total, 390 unique proteins were identified, which included 79 proteins previously annotated as hypothetical or conserved hypothetical, which is the largest number of cell wall and cell wall-associated proteins for mycobacteria reported in one study.

### Hydrophobicity analysis of the identified cell wall proteins

**Figure 1:**
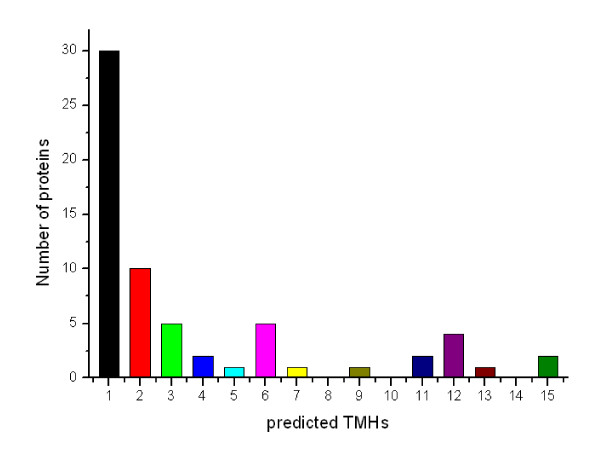
**The distribution of the numbers of identified *M. smegmatis *cell wall proteins for each number of predicted TMHs as predicted by using the TMHMM2.0 program**.

Potential cell wall associated proteins with 1-15 TMHs (Transmembrane helix) were assigned using TMHMM 2.0 program against the *Mycobacterial smegmatis *MC2 155 protein sequence database (excluding the possible signal sequences). In our study, 64 proteins (16.41%) were identified to have at least 1 transmembrane domain. The predicted TMH numbers of these proteins ranged from 1 to 15, and 34 contained at least two TMHs. The profile of TMH in cell wall proteins of *M. smegmatis *is very similar to previous reports about TMH in *M. tuberculosis *cell wall proteome [17]. The distribution of these TMHs is shown in Figure 1. The grand average of hydropathy(GRAVY) value, which is used to evaluate the hydrophilicity and hydrophobicity of a protein along with its amino acid sequence[18], was minus 0.96. There are 21 proteins with GRAVY scores ≥ 0.4, which are so hydrophobic that they are susceptible to precipitation during isoelectric focusing and impossible to be detected by 2-DE. Some important proteins with many TMHs were identified in our study, for example, integral membrane protein MviN and the sugar transport protein including sugar ABC transporter permease protein and sugar transport protein[19]. Apparently, our optimized methods provided a candidate platform that did not appear to be biased against proteins with high hydrophobicity or multiple TMHs.

### Molecular mass and p*I *distributions of the identified cell wall proteins

**Figure 2:**
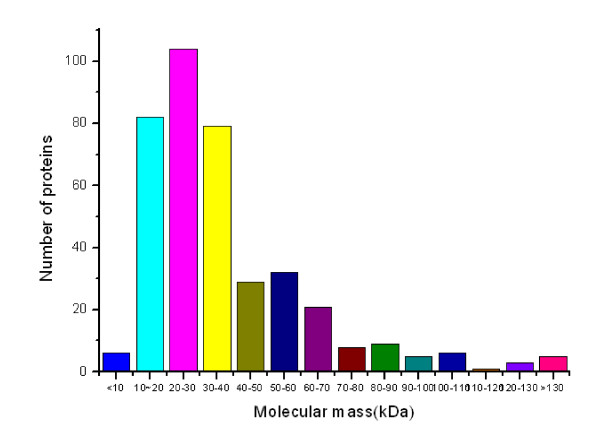
**The distribution of molecular mass (*M*_*r*_) of the total identified *M. smegmatis *cell wall proteins**.

**Figure 3:**
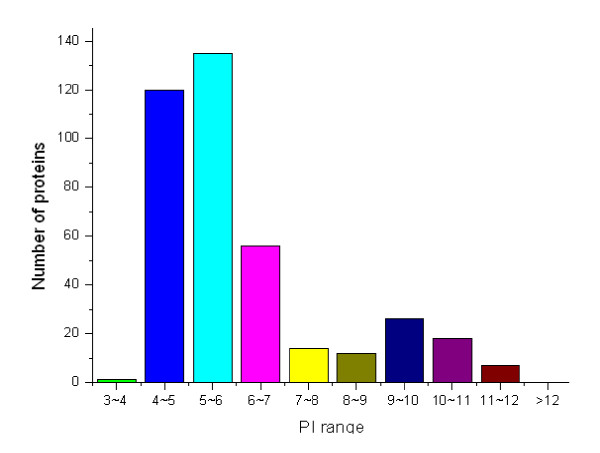
**The distribution of P*I *scores of the total identified *M. smegmatis *cell wall proteins**.

**Figure 4:**
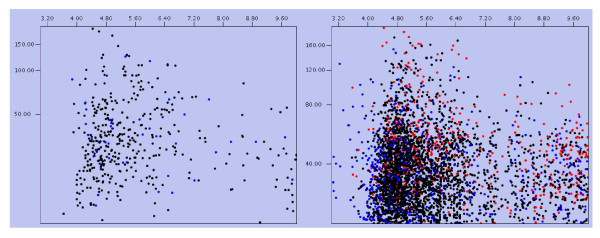
**Virtual 2D-gel of *M. smegmatis *CS2 155**. (A) *M. smegmatis *cell wall proteome; (B) *M. smegmatis *total proteome.

**Figure 5:**
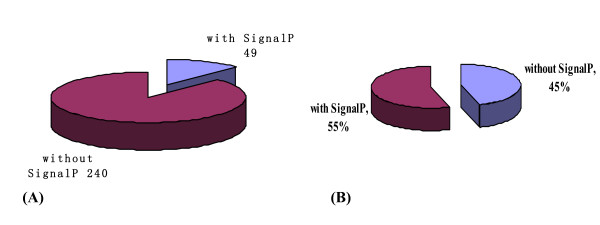
**The distribution of proteins with SignalP in (A) *M. smegmatis *cell wall proteome; (B) *M. smegmatis *cell surface-exposed proteome**.

The theoretical *M*_r _distribution of the identified cell wall proteins ranged from 5.978 kDa to 389.860 kDa. Moreover, proteins between *M*_r _10 and 40 kDa were in the majority, representing approximately 67.95% (265 out of 390) of all the identified cell wall proteins. Detailed distributions are shown in Figure 2. The theoretical p*I *scores of the identified cell wall proteins ranged from 4.16 to 11.56. Detailed distributions are shown in Figure 3. The theoretical p*I *and *M*_r _distribution of the cell wall proteins is demonstrated in a Virtual 2D-gel in Figure 4A. Out of 390 proteins identified, it is obvious that the most proteins clustered around p*I *4-7, and *M*_r _10-40 kDa, which was similar with that of the total proteome (Figure 4B). There are 25 proteins with p*I *scores over 10 and 15 proteins with *M*_r _over 100 kDa. Taking GRAVY value into account, there will be at least 61 (21+25+15) proteins beyond the general 2-DE separation limits. Additionally, there are 49 proteins with predicted signal peptide in the 390 identified cell wall proteins (Figure 5A).

### Analysis of functional groups in identified cell wall protein

**Figure 6:**
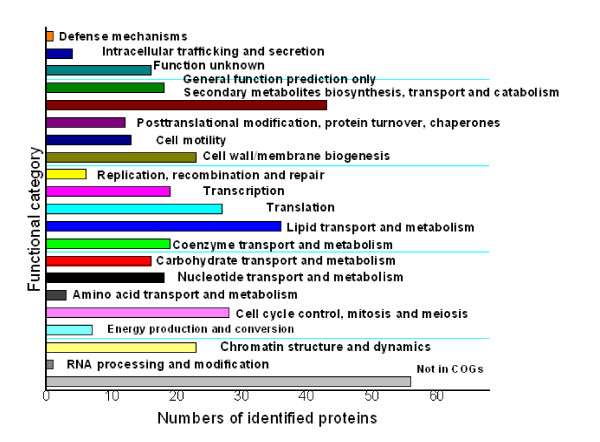
**Functional classification of the identified *M. smegmatis *cell wall proteome**.

Based on the Pasteur Institute functional classification tree http://www.ncbi.nlm.nih.gov/COG/, 390 identified proteins were distributed across twenty one of these functional groups (See table 1 for details). Most of the identified proteins were involved in general function prediction only (functional category R, 11.03%), translation and transcription (16.15%), amino acid transport and metabolism (7.17%), energy production and conversion (5.90%), posttranslational modification, protein turnover, chaperones (5.9%) and replication, recombination and repair (4.87%) (Figure 6). Additionally, 4.62% of the proteins could not be assigned functions in this manner, and 14.36% of the proteins had no related COG. 51.02% of proteins were involved in the six major functional categories above. Many unexpected proteins such as the ribosomal proteins were found to be cell wall associated, which were also found in cell wall by previous research [17,20]. It is probably these proteins interact tightly with the cell wall and join in cell envelop processes and would be potential significance in vaccine studies. Overlap between cytosolic, membrane and cell wall proteins in large scale proteomic studies is not uncommon. Additional studies are necessary to investigate the proteins with multiple cellular locations. The identification of heat-shock proteins in the cell surface exposed fraction might to some extent be due to the strong affinity of these proteins to cell wall proteins. Contact between cytoplasmic and cell surface exposed proteins can not be avoided during the extraction immediately for a brief moment after lysis.

**Table 1:** Functional classification of the identified MC2 155 cell wall proteins

Code	Description	Number
**V**	Defense mechanisms	1
**U**	Intracellular trafficking and secretion	4
**T**	Signal transduction mechanisms	16
**S**	Function unknown	18
**R**	General function prediction only	43
**Q**	Secondary metabolites biosynthesis, transport and catabolism	12
**P**	Inorganic ion transport and metabolism	13
**O**	Posttranslational modification, protein turnover, chaperones	23
**M**	Cell wall/membrane biogenesis	6
**L**	Replication, recombination and repair	19
**K**	Transcription	27
**J**	Translation	36
**I**	Lipid transport and metabolism	19
**H**	Coenzyme transport and metabolism	16
**G**	Carbohydrate transport and metabolism	18
**F**	Nucleotide transport and metabolism	3
**E**	Amino acid transport and metabolism	28
**D**	Cell cycle control, mitosis and meiosis	7
**C**	Energy production and conversion	23
**A**	RNA processing and modification	1
**-**	Not in COGs	56

### Surface exposed proteins

**Figure 7:**
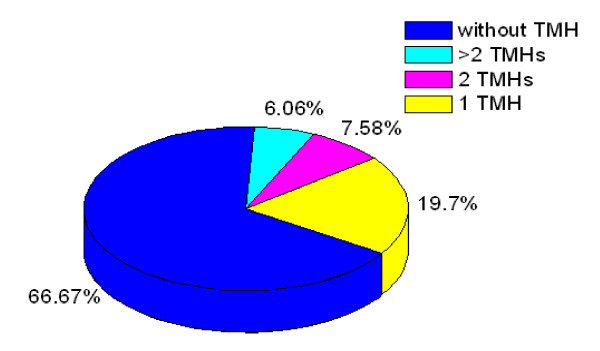
**TMHs of surface exposed proteins of *M. smegmatis *MC2 155**.

**Figure 8:**
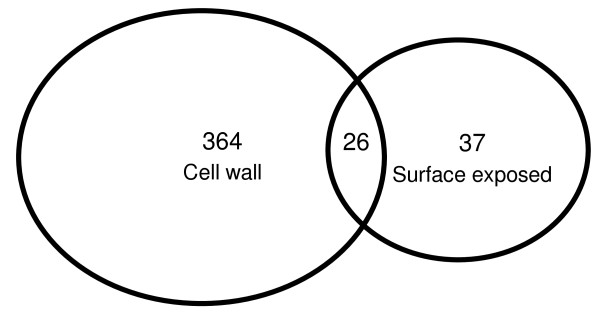
**Venn diagram showing the overlap between cell wall & cell surface exposed proteins**.

Bacterial surface proteins play a fundamental role in the interaction between the bacterial cell and its environment [21-23]. They are involved in adhesion to and invasion of host cells, in sensing the chemical and physical conditions of the external milieu and sending appropriate signals to the cytoplasmic compartment, in mounting defenses against host responses and in toxicity. Therefore, surface exposed proteins are potential targets of drugs aimed at preventing bacterial infections and diseases [24]. Here, to identify the surface-exposed proteins of the *M. smegmatis*, exponentially growing bacteria were collected and treated with trypsin to shave the bacterial surface of exposed protein domains. In previous studies, this 'shaving' proteins technique has resulted in the identification of many surface exposed proteins [20,25]. The integrity of the cells after trypsin treatment was confirmed by viable counts, results of which confirmed the integrity of the cells (as seen in Additional file 2). Peptides released into the supernatant were collected to be fully digested with trypsin for 12~14 h, then concentrated and analyzed by LC-MS/MS. A total of 63 cell surface exposed proteins were successfully identified (as seen in table sup2). The predicted TMH numbers of these proteins ranged from 1 to 3, and 14% of which contained at least two TMHs. The distribution of these TMHs is listed in Figure 7. 55% of the identified proteins have signal peptides (Figure 5B). As seen from Figure 8 that, 26 proteins of 63 found surface-exposed proteins overlapped with the cell wall proteins, which include 11 ribosomal proteins, acyl carrier protein, anion-transporting ATPase, chain A Main Porin, chaperonin GroEL, D-3-phosphoglycerate dehydrogenase, dihydrolipoamide acetyltransferase, DivIVA protein, DNA-directed RNA polymerase subunit beta, elongation factor Tu, enoyl-CoA hydratase, extracellular solute-binding protein family protein 5, glycerol kinase, polyketide synthase, transcription termination factor Rho and trigger factor. The control sample had no protein identified. The discrepancy between the identified surface exposed proteins and the complete cell wall proteome is likely due to the loose association of these proteins with the cell wall which make them prone to detachment. Indeed, some surface proteins are assumed to be attached to the cell wall in a non-covalent way and have been reported to be lost during mild standard manipulations [26,27]. EF-Tu(elongation factor thermo unstable) was identified as a cell wall related protein in this study, which was also been found as cell wall protein in other studies [28]. Translation elongation factors are responsible for two main processes during protein synthesis on the ribosome [29]. EF-Tu is responsible for the selection and binding of the cognate aminoacyl-tRNA to the A-site (acceptor site) of the ribosome. Till now, it is still unclear how proteins such as GroEL, divIVA and elongation factor TU belonging to the unexpected proteins within the *M. smegmatis *cell wall and cell surface exposed proteome leave the bacterial cell, are retained on the cell surface and whether they have an additional function when associated with the cell wall different from their known function inside the bacterial cell.

### Cell division

The proteins related to cell division, divIVA, ftsK, ftsE, ftsX, ftsH and ftsY, were identified as cell wall related proteins in this study. The divIVA gene, which for the most part is confined to gram-positive bacteria, was first identified in *Bacillus subtilis*. Cells with a mutation in this gene have a reduced septation frequency and undergo aberrant polar division, leading to the formation of anucleate minicells [30-32]. The divIVA gene codes for a protein that has been implicated in selection of septum positioning at midcell in vegetative division of *B. subtilis*, where it has been proposed to play a role similar to that of the *E. coli *MinE topological specificity component of the MinCDE division site selection system [33,34]. A divIVA gene is also present in *Streptomyces coelicolor *[35] and in other actinomycetes, like *Mycobacterium tuberculosis*, where Wag31 (antigen 84), a protein proposed to be involved in cell shape maintenance [36]. While many gram-positive bacteria may contain divIVA gene but lack minE and even the full minCDE system, many gram-negative bacteria have minE but no divIV.

FtsE, in association with the integral membrane protein FtsX, is involved in the assembly of potassium ion transport proteins, both of which being relevant to the tubercle bacillus. Recently FtsE and FtsX have been found to localize to the septal ring in *E. coli*, with the localization requiring the cell division proteins FtsZ, FtsA, and ZipA but not FtsK, FtsQ, FtsL, and FtsI proteins [37], suggestive of a role for FtsEX in cell division. Thus, since FtsE of the FtsEX complex shares sequence conservation with ABC type transporter proteins, the complex could be involved in the transport or translocation processes involving drugs, ions, solutes, proteins, peptides or polysaccharides in relation to drug resistance, salt tolerance, cell division or membrane protein insertion.

### Transcriptional regulators

In total, There are 15 transcriptional regulators identified as cell wall related proteins in this work, among which include two ArsR-family proteins, three TetR family proteins and two two-component transcriptional regulatory proteins (detailed information given in Additional file 3). Two-component systems are major elements in bacterial adaptation to environmental changes. These systems are implicated in a large variety of adaptive responses, such as quorum sensing, chemotaxis and metabolic changes. In many pathogenic bacteria, two-component systems are central regulatory elements for the production of virulence factors [38,39]. In this study two two-component transcriptional regulatory proteins, PrrA and DevR were identified in the cell wall proportion. The prrA gene, encoding the regulator of the two-component system PrrA-PrrB, has been shown to be induced upon macrophage phagocytosis and to be transiently required for the early stages of macrophage infection for *M. tuberculosis*[40]. Adaptation to oxygen limitation is likely to constitute a key step in mycobacterial persistence and dormancy and could well be mediated by a two-component system and it is suggested that DevR-DevS might serve as a regulatory link between hypoxia and establishment and/or maintenance of the appropriate response [41].

### Lipid metabolism

The fatty acid components are the most energetically expensive molecules to produce, and thus the regulation of fatty acid production is very tightly controlled to match the growth rate of cells [42]. In this study, proteins related to lipid metabolism, cyclopropane-fatty-acyl-phospholipid synthase 1, fatty acid desaturase, fatty acid synthase, methoxy mycolic acid synthase 1, rhamnolipids biosynthesis 3-oxoacyl-[acyl-carrier-protein] reductase were identified in the cell wall proportion, among which fatty acid synthase and mycolic acid synthase (umaA) play important roles in mycolic acids metabolism. Mycolic acids are important and characteristic constituents of the mycobacterial cell wall. Changes in the structure or composition of mycolic acids have been associated with modification of cell wall permeability and attenuation of pathogenic Mycobacterial strains [43]. Many proteins like fatty acid synthase ACP, related to mycolic acids synthesis and elongation, are considered cell envelope-bound, which was confirmed in this study [44].

### Transport proteins

A cell must selectively translocate molecules across its cell envelop to ensure that the chemical composition of its cytoplasm remains distinct from the surrounding medium [45]. The most important proteins for this purpose are the ABC transporters that actively transport chemically diverse sustrates across the cell wall [46]. The chemical nature of the substrates handled by ABC transporters is extremely diverse from inorganic ions to sugars and large polypeptides; yet ABC transporters are highly conserved. Overexpression of certain ABC transporters is the most frequent cause of resistance to cytotoxic agents including antibiotics, antifungals, herbicides, and anticancer drugs. It is well known that ABC transporters are modular and consist of proteins forming a channel, ATPase components and extracellular-binding proteins where some of these components can be fused together or not [47]. In this study, 28 ABC transporters were identified. Out of these transporters, there were transmembrane proteins with one or six TMHs, and two have signal peptide. These proteins included 12 ATPase components which are predicted to be associated to transmembrane permease of ABC (ATP Binding Cassette) [48,49]. As found by Titgemeyer F. et al, there are 28 putative carbohydrate transporters in *M. smegmatis *and the majority of sugar transport systems (19/28) belong to the ATP-binding cassette (ABC) transporter family [19]. In this study, 10 sugar transport proteins were found in cell wall fraction, and five of which are ABC transporters [19]. Among the ABC transporters identified, ATP binding protein of ABC transporter and ABC transporter periplasmic-binding protein YtfQ, branched-chain amino acid ABC transporter substrate-binding protein, branched-chain amino acid ABC transporter ATP-binding protein are in the same operon respectively.

## Conclusions

We have obtained a comprehensive picture of the *M. smegmatis *cell wall protein repertoire, with an additional insight in the portion of these proteins that are cell surface exposed. With 390 distinct proteins identified, this study represents the first proteomic analysis of cell wall proteins of *M. smegmatis *MC2 155. It also represents the largest number of cell wall and cell wall-associated proteins for mycobacteria reported in one study.

Many of the cell wall-associated proteins appeared to have multiple subcellular localizations. In fact, some proteins previously reported as located in the cytoplasmic compartment were also associated with the bacterial cell wall and cell surface. These proteins supposedly transit between the cytosol and the cell wall compartments, and consequently, their localization, rather than to be strictly compartmentalized, could also depend on physiological and/or environmental conditions. Moreover, their moonlighting role at different subcellular localizations remains to be elucidated in *M. smegmatis*.

## Methods

### Bacterial strain and growth conditions

*M. smegmatis *MC2 155 was grown in Luria Broth (Becton Dickinson, Mississauga, ON, Canada) medium at 37°C with constant agitation (200 rpm) until mid-exponential growth phase. The culture was harvested by centrifugation for 10 min at 10 000 × g at 4°C and washing three times with ice-cold phosphate buffered saline (PBS) (pH7.4). The pelleted cells were frozen at -80°C until needed.

### Cell wall proteins preparation

The extraction of cell wall proteins from *M. smegmatis *MC2 155 was carried out according to Sanjeev et al. with minor modification [50]. Cells from a 1 L culture were harvested at 4400 × g and washed with NaCl solution (0.16 M). The weight of wet cells was determined and for each gram of bacteria one ml lysis buffer (0.05 M potassium phosphate, 0.022% (v/v) β-mercaptoethanol, pH 6.5) was added. Lysozyme (Roche, Mississauga, ON, Canada) was added to the cells to a final concentration of 2.4 mg/ml. The cells were then incubated at 37°C for 2 h. Subsequently, cells (maintained in screw cap Eppendorf tubes) were disrupted with a bead beater (Biospec products, USA) for 4-6 times (1.5 min each time, ice cool down at intervals). The lysates were subjected to a low speed centrifugation at 600 × g to remove unbroken cells. Centrifugation was repeated 3 to 5 times for 40 min at 22,000 × g to pellet the cell walls. All pellets were resuspended and pooled. A second cell lysis the same as before was performed on the pooled pellet. A single centrifugation at 22,000 × g gave the pellet of cell wall fraction. The pellet was resuspended and centrifugated at 22,000 × g, then stored frozen at -80°C.

### Bacterial surface digestion

Procedure was carried out according to Guido Grandi et al [20] with some modifications. Bacteria were harvested from culture at an OD600 of 0.4 (exponential phase) by centrifugation at 3,500 × g for 10 min at 4°C, and washed three times with PBS. Cells were resuspended in one-hundredth volume of PBS containing 40% sucrose (pH 7.4). Digestions were carried out with 20 mg proteomic grade trypsin (Sigma-Aldrich, Oakville, ON, Canada) in the presence of 5 mM DTT, for 30 min at 37°C. A control experiment was carried out in parallel in which we incubated *M. smegmatis *cells in the "trypsin shaving" incubation buffer without trypsin for 2 hours. The digestion mixtures were centrifuged at 3,500 × g for 10 min at 4°C, and the supernatants (Fresh trypsin was added) were incubated at 37°C for around 12~14 hrs for full digestion after being filtered using 0.22 μm pore-size filters (Millipore, Etobicoke, ON, Canada). Protease reactions were stopped with formic acid at 0.1% final concentration. Peptide fractions were concentrated with a Speed-vac centrifuge (Savant), and kept at -20°C until further analysis.

### Sample digestion

Protein sample was separated by 12.5% sodium dodecyl sulfate polyacrylamide gel (SDS-PAGE), run for 1 h at 30 W, then for 4.5 h at 180 W. The gels were Coomassie Brilliant Blue stained and the lane corresponding to the cell wall proteins was cut into 6 equal pieces. The gel pieces were individually in-gel digested as described previously with some modifications [50]. Briefly, after in-gel digestion using trypsin, the digested solution was transferred into a clean 0.6 ml tube. Fifty microliters of 50% acetonitrile (ACN)/5% formic acid (FA) was added to the gel pieces and sonicated for 30 min. This extraction procedure was repeated three times, and a total of 150 μl of extracts was collected. All extracts were pooled and concentrated to less than 10 μl using an SPD 2010 SpeedVac system (Thermo Electron, Waltham, MA). Thereafter, the sample was diluted with 0.1% FA in HPLC water to 100 μL for direct LC-MS/MS analysis or reconstituted with trifluoroacetic acid (TFA) to a final concentration of 0.1% and subjected to sample cleanup steps using C18 ZipTips (Millipore) prior to LC-MS/MS analysis. The C18 ZipTips were conditioned with 100% ACN and then equilibrated three times with 0.1% TFA. The peptides were bound to the ZipTip pipet tip by aspirating and dispensing the sample for at least 15 cycles, washed with 0.1% TFA, and eluted by 20 μL of elution buffer (75% ACN, 0.1% TFA).

### Protein identification by LC-MS/MS

Digests were analyzed using an integrated Agilent 1100 LC-ion-Trap-XCT-Ultra system fitted with an Agilent ChipCube source sprayer. Injected samples were first trapped and desalted on a Zorbax 300 SB-C18 Precolumn (5 μm, 5 × 300-μm inside diameter; Agilent) for 5 min with 0.2% formic acid delivered by the auxiliary pump at 0.3 μl/min. The peptides were then reverse eluted from the trapping column and separated on an analytical Zorbax 15 cm-long 300SB-C18 HPLC-Chip 0.3 μl/min. Peptides were eluted with a 5-45% acetonitrile gradient in 0.2% formic acid over a 50 min interval. Data-dependent acquisition of collision-induced dissociation MS/MS was utilized, and parent ion scans were run over the mass range m/z 400-2,000 at 8,100. For analysis of LC-MS/MS data, Mascot searches used the following parameters: 1.4 Da MS error, 0.8 Da MS/MS error, 1 potential missed cleavage, and variable oxidation (Methionine) [51].

### Protein identification

Data files from the chromatography runs were batch searched against the *M. smegmatis *proteome database using the SEQUEST algorithm16 contained within Bioworks v3.1 software [52]. The criteria used for protein identification were as follows. For positive identification of any individual protein, a minimum of two peptides was required. The minimum cross-correlation coefficients (Xcorr) of 1.9, 2.2, and 3.75 for singly, doubly, and triply charged precursor ions respectively and a minimum ?Cn of 0.1 were both required for individual peptides. For false positive analysis, a decoy search was performed automatically by choosing the Decoy checkbox on the search form.

### Physicochemical characteristics and subcellular localization of the identified proteins

The full set of *M. smegmatis *MC2 155 ORFs was downloaded from the NCBI databases, including 6938 ORFs. The codon adaptation indices (CAI) and hydrophilicity of the proteins were calculated with the standalone version of program CodonW (John Peden, http://bioweb.pasteur.fr/seqanal/interfaces/codonw.html). The hydrophilicity was given as a GRAVY (Grand Average of Hydrophobicity) score [53], which is calculated as the sum of hydropathy values of all the amino acids, divided by the number of residues in the sequence. The TMHMM 2.0 program, based on a hidden Markov model http://www.cbs.dtu.dk/services/TMHMM/, was used to predict protein transmembrane topology [54]. The protein functional family was categorized according to the COG annotation terms http://www.ncbi.nlm.nih.gov/COG/[55]. The virtual 2DE was produced according to Hiller et al. http://www.jvirgel.de/index.html[56].

## Authors' contributions

ZGH carried out the proteomics study, analyzed the data and drafted the manuscript. JDB conceived of the study, and participated in its design and coordination. All authors have read and approved the final manuscript.

## Supplementary Material

Additional file 1 **Cell wall proteins list**. A summarization of all the identified cell wall proteins of *Mycobacterium smegmatis *strain MC2 155. Click here for file

Additional file 2 **Bacterial viable test**. A description of bacterial viable test comparison between cells pretreated with trypsin and control. Click here for file

Additional file 3 **Cell surface-exposed proteins list**. A summarization of all the identified cell surface proteins of *Mycobacterium smegmatis *strain MC2 155. Click here for file
