# Supplementary material for: Cell wall proteome analysis of Mycobacterium smegmatis strain MC2 155
Source: BMC Microbiol. 2010 Apr 22;10:121. doi: 10.1186/1471-2180-10-121 (PMC2867950; doi:10.1186/1471-2180-10-121)
Supplement: Additional file 1 — Cell wall proteins list. A summarization of all the identified cell wall proteins of Mycobacterium smegmatis strain MC2 155. [file 1471-2180-10-121-S1.DOC]

Additional file 1: Cell wall proteins list

| ***Gene ID*** | ***Locus_tag*** | ***Ref_seq*** | ***Locus*** | **Mtb *orthologue*** | ***Protein name*** | **P*I*** | ***MW*** | ***Functional category*** | ***HMMTOP_Helices*** | ***Signal_Details*** |
| --- | --- | --- | --- | --- | --- | --- | --- | --- | --- | --- |
| gi|118467362 | MSMEG_3053 | YP_887369.1 | rpoZ | Rv1390 | DNA-directed RNA polymerase subunit omega [Mycobacterium smegmatis str. MC2 155] | 4.49 | 11695.09 | COG1758K | 0 | No SP detected |
| gi|118467381 | MSMEG_4281 | YP_888557.1 | pepB | Rv2213 | leucyl aminopeptidase [Mycobacterium smegmatis str. MC2 155] | 6.4 | 53725.1 | COG0260E | 0 | No SP detected |
| gi|118467387 | MSMEG_0672 | YP_885081.1 | - | * | hypothetical protein MSMEG_0672 [Mycobacterium smegmatis str. MC2 155] | 7.98 | 23244.41 | - | 0 | No SP detected |
| gi|118467428 | MSMEG_2667 | YP_887005.1 | - | Rv3075c | HpcH/HpaI aldolase/citrate lyase family protein, putative [Mycobacterium smegmatis str. MC2 155] | 4.62 | 33222.16 | COG2301G | 0 | No SP detected |
| gi|118467436 | MSMEG_4917 | YP_889173.1 | - | Rv1324 | TPR repeat-containing protein[Mycobacterium smegmatis str. MC2 155] | 4.59 | 31351.28 | COG3118O | 0 | SP detected |
| gi|118467442 | MSMEG_0639 | YP_885049.1 | - | Rv1281c | oligopeptide transport ATP-binding protein AppF [Mycobacterium smegmatis str. MC2 155] | 6.5 | 36981.41 | COG4608E | 0 | No SP detected |
| gi|118467443 | MSMEG_2062 | YP_886423.1 | nuoB | Rv3146 | NADH dehydrogenase subunit B [Mycobacterium smegmatis str. MC2 155] | 6.8 | 20102.71 | COG0377C | 2 | No SP detected |
| gi|118467472 | MSMEG_0363 | YP_884776.1 | - | Rv0238 | TetR-family protein regulatory protein [Mycobacterium smegmatis str. MC2 155] | 5.29 | 23106.26 | COG1309K | 0 | No SP detected |
| gi|118467483 | MSMEG_2174 | YP_886528.1 | - | Rv3916c | superfamily protein I DNA or RNA helicase [Mycobacterium smegmatis str. MC2 155] | 4.99 | 93891.51 | COG0210L | 0 | No SP detected |
| gi|118467503 | MSMEG_1959 | YP_886325.1 | - | Rv3193c | hypothetical protein MSMEG_1959 [Mycobacterium smegmatis str. MC2 155] | 6.46 | 109093.12 | COG1615S | 7 | No SP detected |
| gi|118467540 | MSMEG_4948 | YP_889200.1 | - | Rv1301 | Sua5/YciO/YrdC/YwlC family protein [Mycobacterium smegmatis str. MC2 155] | 4.71 | 22975.22 | COG0009J | 0 | No SP detected |
| gi|118467545 | MSMEG_2416 | YP_886756.1 | - | Rv2927c | hypothetical protein MSMEG_2416 [Mycobacterium smegmatis str. MC2 155] | 4.72 | 27257.18 | COG3599D | 0 | No SP detected |
| gi|118467565 | MSMEG_2271 | YP_886623.1 | hypB | * | hydrogenase accessory protein HypB [Mycobacterium smegmatis str. MC2 155] | 5.13 | 28389.77 | COG0378OK | 0 | No SP detected |
| gi|118467566 | MSMEG_5055 | YP_889305.1 | - | * | NAD-dependent malic enzyme [Mycobacterium smegmatis str. MC2 155] | 4.97 | 37766.7 | COG0281C | 0 | No SP detected |
| gi|118467576 | MSMEG_0029 | YP_884448.1 | trgG | Rv0013 | para-aminobenzoate synthase component II [Mycobacterium smegmatis str. MC2 155] | 5.47 | 23578.77 | COG0512EH | 1 | No SP detected |
| gi|118467592 | MSMEG_1874 | YP_886240.1 | mtrA | Rv3246c | DNA-binding response regulator MtrA [Mycobacterium smegmatis str. MC2 155] | 5.35 | 25318.38 | COG0745TK | 0 | No SP detected |
| gi|118467594 | MSMEG_1543 | YP_885925.1 | - | Rv0458 | eptc-inducible aldehyde dehydrogenase [Mycobacterium smegmatis str. MC2 155] | 5.01 | 55979.29 | COG1012C | 2 | No SP detected |
| gi|118467595 | MSMEG_6363 | YP_890577.1 | csdA | Rv3778c | cysteine desulfurase family protein [Mycobacterium smegmatis str. MC2 155] | 5.14 | 41904.65 | COG0520E | 0 | No SP detected |
| gi|118467620 | MSMEG_4272 | YP_888549.1 | - | Rv2204c | HesB/YadR/YfhF family protein [Mycobacterium smegmatis str. MC2 155] | 4.4 | 12543.94 | COG0316S | 0 | No SP detected |
| gi|118467630 | MSMEG_1476 | YP_885858.1 | sppA | Rv0724 | SP peptidase SppA, 67K type [Mycobacterium smegmatis str. MC2 155] | 5.26 | 62714.84 | COG0616OU | 4 | No SP detected |
| gi|118467634 | MSMEG_5062 | YP_889312.1 | - | Rv1234 | hypothetical protein MSMEG_5062 [Mycobacterium smegmatis str. MC2 155] | 9.57 | 18604.53 | - | 2 | No SP detected |
| gi|118467664 | MSMEG_4934 | YP_889185.1 | - | Rv1314c | ATP:cob(I)alamin adenosyltransferase [Mycobacterium smegmatis str. MC2 155] | 4.16 | 87347.65 | COG2096S | 1 | No SP detected |
| gi|118467697 | MSMEG_2321 | YP_886667.1 | serB2 | Rv3042c | phosphoserine phosphatase [Mycobacterium smegmatis str. MC2 155] | 4.56 | 37396.51 | COG0560E, COG3830T | 0 | No SP detected |
| gi|118467755 | MSMEG_2440 | YP_886780.1 | rplS | Rv2904c | 50S ribosomal protein L19 [Mycobacterium smegmatis str. MC2 155] | 9.92 | 12867.77 | COG0335J | 0 | No SP detected |
| gi|118467764 | MSMEG_1897 | YP_886263.1 |  | * | 3-oxoadipate enol-lactonase [Mycobacterium smegmatis str. MC2 155] | 5.68 | 27167.71 | COG0596R | 0 | No SP detected |
| gi|118467765 | MSMEG_3328 | YP_887636.1 | - | * | hypothetical protein MSMEG_3328 [Mycobacterium smegmatis str. MC2 155] | 6.2 | 14853.55 | - | 1 | No SP detected |
| gi|118467769 | MSMEG_2329 | YP_886675.1 | - | Rv3038c | methyltransferase, UbiE/COQ5 family protein [Mycobacterium smegmatis str. MC2 155] | 5.01 | 36275.81 | COG0500QR | 0 | No SP detected |
| gi|118467790 | MSMEG_5485 | YP_889723.1 | moaA | Rv0984 | molybdopterin biosynthesis protein [Mycobacterium smegmatis str. MC2 155] | 4.28 | 17944.38 | COG0521H | 0 | No SP detected |
| gi|118467796 | MSMEG_5117 | YP_889365.1 | - | Rv1188 | proline dehydrogenase [Mycobacterium smegmatis str. MC2 155] | 5.56 | 35421.08 | COG0506E | 0 | No SP detected |
| gi|118467809 | MSMEG_2294 | YP_886644.1 | dinP | Rv3056 | DNA polymerase IV [Mycobacterium smegmatis str. MC2 155] | 5.15 | 38492.93 | COG0389L | 0 | No SP detected |
| gi|118467831 | MSMEG_4700 | YP_888961.1 | - | Rv2477c | putative ABC transporter ATP-binding protein [Mycobacterium smegmatis str. MC2 155] | 5.17 | 61893.09 | COG1116P | 1 | No SP detected |
| gi|118467834 | MSMEG_3254 | YP_887565.1 | - | * | RDD family protein, putative [Mycobacterium smegmatis str. MC2 155] | 9.57 | 16922.63 | COG1714S | 3 | No SP detected |
| gi|118467849 | MSMEG_2660 | YP_886997.1 | - | Rv2779c | transcriptional regulator, AsnC family protein [Mycobacterium smegmatis str. MC2 155] | 5.92 | 18577.12 | COG1522K | 1 | No SP detected |
| gi|118467859 | MSMEG_2589 | YP_886926.1 | - | * | hypothetical protein MSMEG_2589 [Mycobacterium smegmatis str. MC2 155] | 6.5 | 14661.65 | COG4954S | 0 | No SP detected |
| gi|118467861 | MSMEG_5430 | YP_889669.1 | - | Rv0068 | retinol dehydrogenase 13 [Mycobacterium smegmatis str. MC2 155] | 9.89 | 31164.44 | COG1028IQR | 0 | No SP detected |
| gi|118467877 | MSMEG_6565 | YP_890777.1 | - | Rv0730 | hypothetical protein MSMEG_6565 [Mycobacterium smegmatis str. MC2 155] | 4.92 | 24535.5 | COG0188L, COG0456R | 0 | No SP detected |
| gi|118467890 | MSMEG_6097 | YP_890318.1 | panC | Rv3602c | pantoate--beta-alanine ligase [Mycobacterium smegmatis str. MC2 155] | 5.57 | 33525.06 | COG0414H | 0 | No SP detected |
| gi|118467898 | MSMEG_0083 | YP_884499.1 | mycP1 | Rv3883c | membrane-anchored mycosin mycp1 [Mycobacterium smegmatis str. MC2 155] | 5.22 | 46453.54 | COG1404O | 4 | SP detected |
| gi|118467900 | MSMEG_6207 | YP_890426.1 | - | * | hypothetical protein MSMEG_6207 [Mycobacterium smegmatis str. MC2 155] | 6.16 | 18662.12 | COG3832S | 0 | No SP detected |
| gi|118467911 | MSMEG_6291 | YP_890509.1 | - | * | D-amino-acid dehydrogenase [Mycobacterium smegmatis str. MC2 155] | 5.39 | 44258.52 | COG0665E | 3 | No SP detected |
| gi|118467920 | MSMEG_4757 | YP_889015.1 | fas | Rv2524c | fatty acid synthase [Mycobacterium smegmatis str. MC2 155] | 4.91 | 329443.27 | COG0304IQ, COG0331I, COG2030I, COG4981I, COG4982I | 0 | No SP detected |
| gi|118467923 | MSMEG_6235 | YP_890454.1 | - | Rv3699 | thiopurine S-methyltransferase (tpmt) superfamily protein [Mycobacterium smegmatis str. MC2 155] | 5.01 | 24500.46 | COG0500QR | 0 | No SP detected |
| gi|118467925 | MSMEG_1046 | YP_885443.1 |  | Rv3041c | ABC-type molybdenum transport system, ATPase component [Mycobacterium smegmatis str. MC2 155] | 5.05 | 30657.62 | COG1119P | 0 | No SP detected |
| gi|118467992 | MSMEG_6929 | YP_891123.1 | - | Rv3910 | integral membrane protein MviN, putative [Mycobacterium smegmatis str. MC2 155] | 5.51 | 125529.56 | COG0515RTKL, COG0728R | 15 | No SP detected |
| gi|118467996 | MSMEG_4703 | YP_888964.1 | plsB2 | Rv2482c | glycerol-3-phosphate acyltransferase [Mycobacterium smegmatis str. MC2 155] | 6.23 | 88937.7 | COG2937I | 2 | No SP detected |
| gi|118468006 | MSMEG_4935 | YP_889187.1 | atpC | Rv1311 | F0F1 ATP synthase subunit epsilon [Mycobacterium smegmatis str. MC2 155] | 4.31 | 13264.8 | COG0355C | 0 | No SP detected |
| gi|118468008 | MSMEG_0402 | YP_884815.1 | nrp | Rv0101 | linear gramicidin synthetase subunit D [Mycobacterium smegmatis str. MC2 155] | 5.27 | 25348.27 | COG1020Q | 0 | No SP detected |
| gi|118468039 | MSMEG_5706 | YP_889939.1 | ercc3 | Rv0861c | DNA or RNA helicase of superfamily protein II [Mycobacterium smegmatis str. MC2 155] | 5.61 | 64743.58 | COG1061KL | 2 | No SP detected |
| gi|118468050 | MSMEG_3738 | YP_888037.1 | engA | Rv1713 | GTP-binding protein EngA [Mycobacterium smegmatis str. MC2 155] | 8.46 | 50140.26 | COG1160R | 0 | No SP detected |
| gi|118468088 | MSMEG_1413 | YP_885796.1 | rocD1 | Rv2322c | ornithine--oxo-acid transaminase [Mycobacterium smegmatis str. MC2 155] | 5.62 | 44688.7 | COG4992E | 1 | No SP detected |
| gi|118468103 | MSMEG_2012 | YP_886376.1 | hisD | Rv1599 | histidinol dehydrogenase [Mycobacterium smegmatis str. MC2 155] | 5.01 | 46874.96 | COG0141E | 0 | No SP detected |
| gi|118468129 | MSMEG_1367 | YP_885753.1 | rpoB | Rv0668 | DNA-directed RNA polymerase subunit beta [Mycobacterium smegmatis str. MC2 155] | 5.33 | 148152.54 | COG0085K | 1 | No SP detected |
| gi|118468162 | MSMEG_3149 | YP_887464.1 | - | Rv1481 | hypothetical protein MSMEG_3149 [Mycobacterium smegmatis str. MC2 155] | 7.94 | 35949.65 | COG2304R | 5 | No SP detected |
| gi|118468181 | MSMEG_4248 | YP_888525.1 |  | Rv2182c | 1-acylglycerol-3-phosphate O-acyltransferase [Mycobacterium smegmatis str. MC2 155] | 9.2 | 26378.66 | COG0204I | 1 | No SP detected |
| gi|118468245 | MSMEG_6106 | YP_890327.1 | ephA | Rv3617 | epoxide hydrolase [Mycobacterium smegmatis str. MC2 155] | 4.9 | 35123.44 | COG0596R | 0 | No SP detected |
| gi|118468301 | MSMEG_1523 | YP_885905.1 | rpsD | Rv3458c | 30S ribosomal protein S4 [Mycobacterium smegmatis str. MC2 155] | 10.1 | 23375.73 | COG0522J | 0 | No SP detected |
| gi|118468311 | MSMEG_5511 | YP_889748.1 | - | Rv0959 | von Willebrand factor, type A [Mycobacterium smegmatis str. MC2 155] | 5.87 | 74635.69 | COG4867R | 0 | No SP detected |
| gi|118468312 | MSMEG_2753 | YP_887084.1 | - | Rv2709 | hypothetical protein MSMEG_2753 [Mycobacterium smegmatis str. MC2 155] | 9.15 | 16044.54 | COG0601EP | 2 | No SP detected |
| gi|118468333 | MSMEG_5073 | YP_889323.1 | - | Rv1220c | O-methyltransferase, family protein 3 [Mycobacterium smegmatis str. MC2 155] | 4.75 | 21703.5 | COG4122R | 0 | No SP detected |
| gi|118468354 | MSMEG_1350 | YP_885737.1 | cmaA1 | Rv3392c | cyclopropane-fatty-acyl-phospholipid synthase 1 [Mycobacterium smegmatis str. MC2 155] | 4.85 | 32591.11 | COG2230M | 0 | No SP detected |
| gi|118468356 | MSMEG_0692 | YP_885100.1 | - | Rv0312 | hypothetical protein MSMEG_0692 [Mycobacterium smegmatis str. MC2 155] | 4.23 | 59554.16 | COG0443O | 1 | No SP detected |
| gi|118468357 | MSMEG_2606 | YP_886943.1 |  | * | ArsR-family protein transcriptional regulator [Mycobacterium smegmatis str. MC2 155] | 6.4 | 11198.73 | COG0640K | 0 | No SP detected |
| gi|118468367 | MSMEG_2430 | YP_886770.1 | ffh | Rv2916c | signal recognition particle protein [Mycobacterium smegmatis str. MC2 155] | 8.87 | 54765.32 | COG0541U | 0 | No SP detected |
| gi|118468368 | MSMEG_1416 | YP_885799.1 | - | Rv0688 | Pyridine nucleotide-disulphide oxidoreductase [Mycobacterium smegmatis str. MC2 155] | 4.83 | 41922.42 | COG0446R | 0 | No SP detected |
| gi|118468388 | MSMEG_0927 | YP_885330.1 | - | Rv0481c | hypothetical protein MSMEG_0927 [Mycobacterium smegmatis str. MC2 155] | 4.73 | 18158.41 | - | 0 | No SP detected |
| gi|118468406 |  | YP_888758.1 | - | Rv2358 | transcriptional regulator, ArsR family protein [Mycobacterium smegmatis str. MC2 155] | 6.1 | 12472.24 | COG0640K | 0 | No SP detected |
| gi|118468476 | MSMEG_3748 | YP_888046.1 | - | Rv1697 | thiamin pyrophosphokinase, catalytic domain protein [Mycobacterium smegmatis str. MC2 155] | 5.79 | 42294.62 | COG4825S | 1 | No SP detected |
| gi|118468477 | MSMEG_1557 | YP_885937.1 | rpsI | Rv3442c | ribosomal protein S9 [Mycobacterium smegmatis str. MC2 155] | 10.04 | 16765.35 | COG0103J | 0 | No SP detected |
| gi|118468481 | MSMEG_1344 | YP_885731.1 | secE | Rv0638 | preprotein translocase subunit SecE [Mycobacterium smegmatis str. MC2 155] | 9.3 | 15286.45 | COG0690U | 1 | No SP detected |
| gi|118468511 | MSMEG_4240 | YP_888517.1 | idsA2 | Rv2173 | polyprenyl synthetase [Mycobacterium smegmatis str. MC2 155] | 4.92 | 38775.55 | COG0142H | 0 | No SP detected |
| gi|118468542 | MSMEG_2346 | YP_886690.1 | - | * | phytoene synthase [Mycobacterium smegmatis str. MC2 155] | 6.06 | 35250.03 | COG1562I | 0 | No SP detected |
| gi|118468558 | MSMEG_4497 | YP_888769.1 | phoH1 | Rv2368c | PhoH family protein [Mycobacterium smegmatis str. MC2 155] | 5.67 | 37520.8 | COG1702T | 0 | No SP detected |
| gi|118468621 | MSMEG_1567 | YP_885946.1 | - | Rv3438 | hypothetical protein MSMEG_1567 [Mycobacterium smegmatis str. MC2 155] | 8.86 | 29289.6 | - | 0 | No SP detected |
| gi|118468633 | MSMEG_2259 | YP_886611.1 | cstA | Rv3063 | carbon starvation protein A [Mycobacterium smegmatis str. MC2 155] | 8.66 | 81946.47 | COG1966T | 16 | No SP detected |
| gi|118468680 | MSMEG_1981 | YP_886345.1 | - | * | hypothetical protein MSMEG_1981 [Mycobacterium smegmatis str. MC2 155] | 11.36 | 18176.24 | - | 0 | No SP detected |
| gi|118468684 | MSMEG_4108 | YP_888390.1 | pntB | Rv0157 | NAD(P) transhydrogenase, beta subunit [Mycobacterium smegmatis str. MC2 155] | 6.81 | 50325.48 | COG1282C | 9 | No SP detected |
| gi|118468686 | MSMEG_1957 | YP_886323.1 | - | Rv3195 | hypothetical protein MSMEG_1957 [Mycobacterium smegmatis str. MC2 155] | 4.48 | 49397.52 | COG5282S | 0 | No SP detected |
| gi|118468714 | MSMEG_2410 | YP_886750.1 | - | Rv2969c | putative serine-threonine protein kinase [Mycobacterium smegmatis str. MC2 155] | 5.57 | 25570.16 | COG1651O | 1 | No SP detected |
| gi|118468738 | MSMEG_4959 | YP_889211.1 | argS | Rv1292 | arginyl-tRNA synthetase [Mycobacterium smegmatis str. MC2 155] | 5.06 | 59470.91 | COG0018J | 1 | No SP detected |
| gi|118468744 | MSMEG_6398 | YP_890611.1 | fbpA | Rv3804c | antigen 85-A [Mycobacterium smegmatis str. MC2 155] | 6.18 | 35701.38 | COG0627R | 1 | No SP detected |
| gi|118468773 | MSMEG_0967 | YP_885369.1 | - | Rv0523c | hypothetical protein MSMEG_0967 [Mycobacterium smegmatis str. MC2 155] | 11.52 | 14383.76 | - | 0 | No SP detected |
| gi|118468781 | MSMEG_6411 | YP_890624.1 | - | Rv3819 | hypothetical protein MSMEG_6411 [Mycobacterium smegmatis str. MC2 155] | 9.68 | 12668.48 | - | 0 | No SP detected |
| gi|118468790 | MSMEG_4271 | YP_888548.1 | - | Rv2203 | hypothetical protein MSMEG_4271 [Mycobacterium smegmatis str. MC2 155] | 5.48 | 23501.69 | - | 1 | No SP detected |
| gi|118468795 | MSMEG_2540 | YP_886877.1 | yrH | Rv2883c | uridylate kinase [Mycobacterium smegmatis str. MC2 155] | 6.15 | 26363.46 | COG0528F | 0 | No SP detected |
| gi|118468808 | MSMEG_3645 | YP_887948.1 | - | Rv1829 | hypothetical protein MSMEG_3645 [Mycobacterium smegmatis str. MC2 155] | 4.47 | 18114.52 | COG1259S | 0 | No SP detected |
| gi|118468824 | MSMEG_3770 | YP_888064.1 | argG | Rv1658 | argininosuccinate synthase [Mycobacterium smegmatis str. MC2 155] | 5.3 | 43968.56 | COG0137E | 0 | No SP detected |
| gi|118468859 | MSMEG_0251 | YP_884666.1 | - | Rv0207c | hypothetical protein MSMEG_0251 [Mycobacterium smegmatis str. MC2 155] | 4.49 | 25249.46 | - | 0 | No SP detected |
| gi|118468917 | MSMEG_5871 | YP_890097.1 | - | Rv0759c | HIT family protein [Mycobacterium smegmatis str. MC2 155] | 4.85 | 15618.93 | COG0537FGR | 0 | No SP detected |
| gi|118468918 | MSMEG_2486 | YP_886825.1 | - | Rv1200 | major facilitator superfamily protein [Mycobacterium smegmatis str. MC2 155] | 6.54 | 54212.29 | COG0366G | 12 | No SP detected |
| gi|118468923 | MSMEG_2695 | YP_887030.1 |  | Rv2744c | 35 kDa protein [Mycobacterium smegmatis str. MC2 155] | 5.55 | 30338.07 | COG1842KT | 0 | No SP detected |
| gi|118468948 | MSMEG_4707 | YP_888967.1 | - | * | non-heme bromoperoxidase BPO-A2 [Mycobacterium smegmatis str. MC2 155] | 4.9 | 30161.98 | COG0596R | 0 | No SP detected |
| gi|118468956 | MSMEG_6195 | YP_890414.1 | - | Rv3680 | ion-transporting ATPase [Mycobacterium smegmatis str. MC2 155] | 5.21 | 40339.26 | COG0003P | 1 | No SP detected |
| gi|118468974 | MSMEG_1477 | YP_885859.1 | kgtP | Rv3476c | major facilitator superfamily protein [Mycobacterium smegmatis str. MC2 155] | 5 | 50841.33 | COG0477GEPR | 12 | No SP detected |
| gi|118468979 | MSMEG_6065 | YP_890287.1 | rpsR | Rv0055 | 30S ribosomal protein S18 [Mycobacterium smegmatis str. MC2 155] | 10.61 | 9516.1 | COG0238J | 0 | No SP detected |
| gi|118468983 | MSMEG_2964 | YP_887283.1 | apt | Rv2584c | adenine phosphoribosyltransferase [Mycobacterium smegmatis str. MC2 155] | 5.13 | 18385.14 | COG0503F | 0 | No SP detected |
| gi|118468988 | MSMEG_3221 | YP_887535.1 | trpA | Rv1613 | tryptophan synthase subunit alpha [Mycobacterium smegmatis str. MC2 155] | 4.58 | 27073.57 | COG0159E | 0 | No SP detected |
| gi|118469004 | MSMEG_6402 | YP_890615.1 | - | Rv3807c | PAP2 superfamily protein [Mycobacterium smegmatis str. MC2 155] | 11.32 | 17974.79 | COG0671I | 0 | No SP detected |
| gi|118469010 | MSMEG_4479 | YP_888751.1 | - | Rv2342 | hypothetical protein MSMEG_4479 [Mycobacterium smegmatis str. MC2 155] | 10.89 | 9205.62 | - | 0 | No SP detected |
| gi|118469023 | MSMEG_6189 | YP_890409.1 | - | Rv3676 | transcriptional regulator, Crp/Fnr family protein [Mycobacterium smegmatis str. MC2 155] | 9.57 | 24775.34 | COG0664T | 0 | No SP detected |
| gi|118469037 | MSMEG_1803 | YP_886175.1 | rsbW | Rv3287c | RsbW protein [Mycobacterium smegmatis str. MC2 155] | 4.3 | 14677.63 | - | 0 | No SP detected |
| gi|118469038 | MSMEG_6277 | YP_890496.1 | cobQ2 | Rv3713 | cobyric acid synthase [Mycobacterium smegmatis str. MC2 155] | 5.65 | 25402.83 | COG3442R | 0 | No SP detected |
| gi|118469115 | MSMEG_1441 | YP_885824.1 | rplV | Rv0706 | 50S ribosomal protein L22 [Mycobacterium smegmatis str. MC2 155] | 10.95 | 16323.57 | COG0091J | 0 | No SP detected |
| gi|118469118 | MSMEG_2628 | YP_886965.1 | infB | Rv2839c | translation initiation factor IF-2 [Mycobacterium smegmatis str. MC2 155] | 4.7 | 65927.23 | COG0532J | 0 | No SP detected |
| gi|118469120 | MSMEG_0538 | YP_884949.1 | - | * | regulatory protein, MarR [Mycobacterium smegmatis str. MC2 155] | 6.97 | 17766.73 | COG1846K | 0 | No SP detected |
| gi|118469122 | MSMEG_2758 | YP_887090.1 | SigA | Rv2703 | RNA polymerase sigma factor [Mycobacterium smegmatis str. MC2 155] | 4.72 | **57791** | COG0568K | 0 | No SP detected |
| gi|118469178 | MSMEG_4284 | YP_888561.1 | - | Rv2216 | hypothetical protein MSMEG_4284 [Mycobacterium smegmatis str. MC2 155] | 6.6 | 31810.32 | COG1090R | 1 | No SP detected |
| gi|118469179 | MSMEG_0062 | YP_884480.1 | - | Rv3871 | ftsk/SpoIIIE family protein [Mycobacterium smegmatis str. MC2 155] | 6.18 | 64560.83 | COG1674D | 0 | No SP detected |
| gi|118469186 | MSMEG_5573 | YP_889808.1 | - | * | sugar ABC transporter permease protein [Mycobacterium smegmatis str. MC2 155] | 8.01 | 35393.79 | COG1175G | 6 | No SP detected |
| gi|118469191 | MSMEG_5307 | YP_889553.1 | - | * | TetR-family protein transcriptional regulator [Mycobacterium smegmatis str. MC2 155] | 5.72 | 25529.92 | COG1309K | 0 | No SP detected |
| gi|118469194 | MSMEG_4939 | YP_889191.1 | atpH | Rv1307 | ATP synthase delta chain [Mycobacterium smegmatis str. MC2 155] | 5.07 | 47449.89 | COG0711C, | 1 | No SP detected |
| gi|118469222 | MSMEG_2528 | YP_886865.1 | - | Rv2205c | glycerate kinase [Mycobacterium smegmatis str. MC2 155] | 4.49 | 37697.97 | COG1929G | 0 | No SP detected |
| gi|118469231 | MSMEG_3897 | YP_888187.1 | - | Rv2112c | proteasome component [Mycobacterium smegmatis str. MC2 155] | 5.19 | 54573.67 | - | 1 | No SP detected |
| gi|118469244 | MSMEG_5677 | YP_889910.1 | - | * | hypothetical protein MSMEG_5677 [Mycobacterium smegmatis str. MC2 155] | 4.57 | 20656.29 | - | 0 | No SP detected |
| gi|118469247 | MSMEG_6906 | YP_891105.1 | - | Rv0045c | putative hydrolase [Mycobacterium smegmatis str. MC2 155] | 5.37 | 33317.55 | COG0596R | 0 | No SP detected |
| gi|118469254 | MSMEG_2351 | YP_886694.1 | etfB/ fixA | Rv3029c | electron transfer flavoprotein, beta subunit [Mycobacterium smegmatis str. MC2 155] | 4.66 | 28080.79 | COG2086C | 0 | No SP detected |
| gi|118469262 | MSMEG_2275 | YP_886627.1 | hypD | * | hydrogenase expression/formation protein HypD [Mycobacterium smegmatis str. MC2 155] | 5.56 | 39817.58 | COG0409O | 0 | No SP detected |
| gi|118469266 | MSMEG_6173 | YP_890393.1 | - | Rv3661 | morphological differentiation-associated protein [Mycobacterium smegmatis str. MC2 155] | 5.56 | 31496.95 | COG0560E | 1 | No SP detected |
| gi|118469285 | MSMEG_4916 | YP_889171.1 | glgE | Rv1327c | alpha-amylase family protein [Mycobacterium smegmatis str. MC2 155] | 5.64 | 78043.41 | COG0366G | 0 | No SP detected |
| gi|118469318 | MSMEG_0092 | YP_884508.1 | - | Rv0144 | probable transcriptional regulatory protein [Mycobacterium smegmatis str. MC2 155] | 5.35 | 23067.25 | COG1309K | 1 | No SP detected |
| gi|118469340 | MSMEG_0260 | YP_884675.1 | - | * | DNA-binding protein [Mycobacterium smegmatis str. MC2 155] | 6.24 | 29674.8 | COG1396K | 0 | No SP detected |
| gi|118469347 | MSMEG_5136 | YP_889382.1 | - | * | helix-turn-helix motif [Mycobacterium smegmatis str. MC2 155] | 6.07 | 16673.91 | COG3467R | 0 | No SP detected |
| gi|118469383 | MSMEG_5228 | YP_889474.1 |  | Rv1106c | 3-beta hydroxysteroid dehydrogenase/isomerase family protein [Mycobacterium smegmatis str. MC2 155] | 7.16 | 38805.31 | COG0451MG | 0 | No SP detected |
| gi|118469425 | MSMEG_1930 | YP_886296.1 | rhlE | Rv3211 | DEAD/DEAH box helicase [Mycobacterium smegmatis str. MC2 155] | 6.3 | 54716.54 | COG0513LKJ | 0 | No SP detected |
| gi|118469435 | MSMEG_6896 | YP_891096.1 | ssb | Rv0054 | single-strand DNA-binding protein [Mycobacterium smegmatis str. MC2 155] | 5.12 | 17353.11 | COG0629L | 0 | No SP detected |
| gi|118469452 | MSMEG_0121 | YP_884537.1 | - | Rv1928c | rhamnolipids biosynthesis 3-oxoacyl-[acyl-carrier-protein] reductase [Mycobacterium smegmatis str. MC2 155] | 6.09 | 26312.07 | COG1028IQR | 0 | No SP detected |
| gi|118469477 | MSMEG_2776 | YP_887107.1 | dxs1 | Rv2682c | 1-deoxy-D-xylulose-5-phosphate synthase [Mycobacterium smegmatis str. MC2 155] | 5.7 | 68035.26 | COG1154HI | 2 | No SP detected |
| gi|118469485 | MSMEG_3536 | YP_887839.1 | - | * | sugar transport protein [Mycobacterium smegmatis str. MC2 155] | 7.01 | 66845.17 | COG0477GEPR | 12 | No SP detected |
| gi|118469496 | MSMEG_5773 | YP_890001.1 | desA1 | Rv0824c | fatty acid desaturase [Mycobacterium smegmatis str. MC2 155] | 5.07 | 38454.38 | - | 1 | No SP detected |
| gi|118469498 | MSMEG_5776 | YP_890005.1 | phoU | Rv0821c | phosphate transport system regulatory protein PhoU [Mycobacterium smegmatis str. MC2 155] | 5.14 | 24635.02 | COG0704P | 0 | No SP detected |
| gi|118469510 | MSMEG_0880 | YP_885283.1 | groEL | Rv0440 | chaperonin GroEL [Mycobacterium smegmatis str. MC2 155] | 4.81 | 56487.29 | COG0459O | 0 | No SP detected |
| gi|118469529 | MSMEG_4671 | YP_888934.1 | clpX | Rv2457c | ATP-dependent protease ATP-binding subunit [Mycobacterium smegmatis str. MC2 155] | 5.09 | 46675.56 | COG1219O | 1 | No SP detected |
| gi|118469532 | MSMEG_3124 | YP_887439.1 | sufC | Rv1463 | FeS assembly ATPase SufC [Mycobacterium smegmatis str. MC2 155] | 5.21 | 27561.25 | COG0396O | 0 | No SP detected |
| gi|118469541 | MSMEG_3839 | YP_888130.1 | polA | Rv1629 | DNA polymerase I [Mycobacterium smegmatis str. MC2 155] | 4.95 | 99897.68 | COG0258L, | 1 | No SP detected |
| gi|118469551 | MSMEG_0387 | YP_884800.1 | Rmt2 | * | Rmt2 protein [Mycobacterium smegmatis str. MC2 155] | 7.2 | 34721.53 | COG0500QR | 0 | No SP detected |
| gi|118469556 | MSMEG_1524 | YP_885906.1 | rpoA | Rv3457c | DNA-directed RNA polymerase subunit alpha [Mycobacterium smegmatis str. MC2 155] | 4.63 | 36512.55 | COG0202K | 0 | No SP detected |
| gi|118469591 | MSMEG_6392 | YP_890605.1 | pks13 | Rv3800c | polyketide synthase [Mycobacterium smegmatis str. MC2 155] | 4.6 | 194466.91 | COG3319Q, COG3321Q | 0 | No SP detected |
| gi|118469641 | MSMEG_1472 | YP_885854.1 | rpsE | Rv0721 | 30S ribosomal protein S5 [Mycobacterium smegmatis str. MC2 155] | 10.17 | 21911.02 | COG0098J | 0 | No SP detected |
| gi|118469645 | MSMEG_5070 | YP_889320.1 | htrA | Rv1223 | Trypsin [Mycobacterium smegmatis str. MC2 155] | 4.79 | 51631.97 | COG0265O | 1 | No SP detected |
| gi|118469649 | MSMEG_3146 | YP_887461.1 | - | Rv1478 | invasin 1 [Mycobacterium smegmatis str. MC2 155] | 9.79 | 24672.05 | COG0791M | 1 | SP detected |
| gi|118469679 | MSMEG_1679 | YP_886055.1 | amiB1 | Rv3306c | AmiB [Mycobacterium smegmatis str. MC2 155] | 5.39 | 40728.85 | COG1473R | 0 | No SP detected |
| gi|118469696 | MSMEG_6076 | YP_890298.1 | ispD | Rv3582c | 2-C-methyl-D-erythritol 4-phosphate cytidylyltransferase [Mycobacterium smegmatis str. MC2 155] | 5.49 | 23797.31 | COG1211I | 0 | No SP detected |
| gi|118469768 | MSMEG_1368 | YP_885754.1 | rpoc | Rv0668 | DNA-directed RNA polymerase subunit beta' [Mycobacterium smegmatis str. MC2 155] | 5.33 | 148152.54 | COG0085K | 1 | No SP detected |
| gi|118469816 | MSMEG_3246 | YP_887557.1 | - | Rv1626 | response regulator [Mycobacterium smegmatis str. MC2 155] | 4.92 | 23173.61 | COG3707T | 0 | No SP detected |
| gi|118469817 | MSMEG_1705 | YP_886081.1 | - | * | D-xylose transport ATP-binding protein XylG [Mycobacterium smegmatis str. MC2 155] | 6.19 | 55661.9 | COG1129G | 0 | No SP detected |
| gi|118469832 | MSMEG_6153 | YP_890373.1 | dinB | Rv3644c | DNA polymerase III subunit delta' [Mycobacterium smegmatis str. MC2 155] | 8.1 | 41784.51 | COG0470L | 0 | No SP detected |
| gi|118469839 | MSMEG_3178 | YP_887493.1 | dnaE | Rv1547 | DNA polymerase III subunit alpha [Mycobacterium smegmatis str. MC2 155] | 5.5 | 129207.26 | COG0587L | 0 | No SP detected |
| gi|118469844 | MSMEG_6219 | YP_890438.1 | moxR2 | Rv3692 | ATPase family protein associated with various cellular activities (AAA) [Mycobacterium smegmatis str. MC2 155] | 5.63 | 34777 | COG0714R | 0 | No SP detected |
| gi|118469870 | MSMEG_1133 | YP_885527.1 | grcC1 | Rv0562 | bifunctional short chain isoprenyl diphosphate synthase [Mycobacterium smegmatis str. MC2 155] | 4.68 | 35410.8 | COG0142H | 0 | No SP detected |
| gi|118469876 | MSMEG_6385 | YP_890598.1 | dltE | Rv3791 | short chain dehydrogenase [Mycobacterium smegmatis str. MC2 155] | 7.85 | 27191.67 | COG1028IQR | 3 | No SP detected |
| gi|118469882 | MSMEG_2788 | YP_887119.1 | - | Rv2670c | ATP/GTP-binding integral membrane protein [Mycobacterium smegmatis str. MC2 155] | 5.54 | 39659.24 | COG1485R | 2 | No SP detected |
| gi|118469884 | MSMEG_1424 | YP_885807.1 | lldD1 | Rv0694 | FMN-dependent dehydrogenase [Mycobacterium smegmatis str. MC2 155] | 5.85 | 42774.07 | COG1304C | 0 | No SP detected |
| gi|118469888 | MSMEG_4210 | YP_888487.1 | - | * | secreted protein [Mycobacterium smegmatis str. MC2 155] | 4.61 | 34566.27 | COG3181S | 3 | No SP detected |
| gi|118469915 | MSMEG_2519 | YP_886856.1 | rpsB | Rv2890c | 30S ribosomal protein S2 [Mycobacterium smegmatis str. MC2 155] | 5.54 | 31761.07 | COG0052J | 1 | No SP detected |
| gi|118469921 | MSMEG_6759 | YP_890967.1 | glpK | Rv3696c | glycerol kinase [Mycobacterium smegmatis str. MC2 155] | 4.74 | 55098.4 | COG0554C | 0 | No SP detected |
| gi|118469964 | MSMEG_6285 | YP_890504.1 | dnaZX | Rv3721c | DNA polymerase III subunits gamma and tau [Mycobacterium smegmatis str. MC2 155] | 5.14 | 69889.35 | COG2812L | 0 | No SP detected |
| gi|118469973 | MSMEG_5721 | YP_889954.1 | fadA | Rv0859 | acetyl-CoA acetyltransferase [Mycobacterium smegmatis str. MC2 155] | 5.03 | 42596.4 | COG0183I | 1 | No SP detected |
| gi|118469982 | MSMEG_0174 | YP_884590.1 | - | * | putative inner membrane protein [Mycobacterium smegmatis str. MC2 155] | 11.08 | 13002.53 | COG2149S | 3 | No SP detected |
| gi|118469989 | MSMEG_4207 | YP_888484.1 | - | * | universal stress protein family protein [Mycobacterium smegmatis str. MC2 155] | 5.27 | 13959.97 | COG0589T | 0 | No SP detected |
| gi|118469997 | MSMEG_3147 | YP_887462.1 | moxR | Rv1479 | ATPase, MoxR family protein [Mycobacterium smegmatis str. MC2 155] | 5.08 | 36795.37 | COG0714R | 0 | No SP detected |
| gi|118469999 | MSMEG_4401 | YP_888676.1 | - | * | phosphonoacetaldehyde hydrolase [Mycobacterium smegmatis str. MC2 155] | 4.9 | 25117.29 | COG0546R | 0 | No SP detected |
| gi|118470004 | MSMEG_2276 | YP_886628.1 | hypE | * | hydrogenase expression/formation protein HypE [Mycobacterium smegmatis str. MC2 155] | 4.87 | 36536.71 | COG0309O | 0 | No SP detected |
| gi|118470039 | MSMEG_0732 | YP_885138.1 | clpB | Rv0384c | chaperone ClpB [Mycobacterium smegmatis str. MC2 155] | 5.04 | 92897.51 | COG0542O | 0 | No SP detected |
| gi|118470092 | MSMEG_0394 | YP_884807.1 | - | * | hypothetical protein MSMEG_0394 [Mycobacterium smegmatis str. MC2 155] | 8.25 | 9194.77 | - | 0 | No SP detected |
| gi|118470102 | MSMEG_3743 | YP_888042.1 | soj | Rv1708 | SpoOJ regulator protein [Mycobacterium smegmatis str. MC2 155] | 6.71 | 38669.45 | COG1192D | 1 | No SP detected |
| gi|118470129 | MSMEG_6223 | YP_890442.1 | - | * | TetR family protein transcriptional repressor LfrR [Mycobacterium smegmatis str. MC2 155] | 5.35 | 20618.45 | COG1309K | 0 | No SP detected |
| gi|118470135 | MSMEG_3890 | YP_888180.1 | - | Rv2097c | proteasome component [Mycobacterium smegmatis str. MC2 155] | 6.47 | 38117.06 | - | 0 | No SP detected |
| gi|118470140 | MSMEG_6935 | YP_891129.1 | - | Rv3915 | N-acetylmuramoyl-L-alanine amidase [Mycobacterium smegmatis str. MC2 155] | 6.61 | 42862.37 | COG0860M, COG3409M | 0 | No SP detected |
| gi|118470162 | MSMEG_6897 | YP_891097.1 | rpsF | Rv0053 | 30S ribosomal protein S6 [Mycobacterium smegmatis str. MC2 155] | 6.09 | 10184.69 | COG0360J | 0 | No SP detected |
| gi|118470172 | MSMEG_3059 | YP_887375.1 | lipI | Rv1400c | esterase [Mycobacterium smegmatis str. MC2 155] | 4.72 | 30968.57 | COG0657I | 0 | No SP detected |
| gi|118470189 | MSMEG_6518 | YP_890731.1 | - | Rv3863 | hypothetical protein MSMEG_6518 [Mycobacterium smegmatis str. MC2 155] | 5.03 | 13362.22 | - | 0 | No SP detected |
| gi|118470225 | MSMEG_1465 | YP_885847.1 | rplN | Rv0714 | ribosomal protein L14 [Mycobacterium smegmatis str. MC2 155] | 10.23 | 13290.48 | COG0093J | 0 | No SP detected |
| gi|118470234 | MSMEG_5042 | YP_889292.1 | deaD | Rv1253 | ATP-dependent rna helicase, dead/deah box family protein [Mycobacterium smegmatis str. MC2 155] | 7.29 | 63723.41 | COG0513LKJ | 0 | No SP detected |
| gi|118470242 | MSMEG_3063 | YP_887379.1 | - | * | LemA protein [Mycobacterium smegmatis str. MC2 155] | 6.4 | 19062.7 | COG1704S | 2 | No SP detected |
| gi|118470244 | MSMEG_6403 | YP_890616.1 | glfT | Rv3808c | bifunctional udp-galactofuranosyl transferase glft [Mycobacterium smegmatis str. MC2 155] | 6.4 | 71572.56 | COG1216R | 0 | No SP detected |
| gi|118470249 | MSMEG_0900 | YP_885303.1 | - | Rv0458 | eptc-inducible aldehyde dehydrogenase [Mycobacterium smegmatis str. MC2 155] | 4.93 | 54769.83 | COG1012C | 2 | No SP detected |
| gi|118470253 | MSMEG_6186 | YP_890406.1 | - | Rv3673c | hypothetical protein MSMEG_6186 [Mycobacterium smegmatis str. MC2 155] | 4.57 | 22738.89 | COG0526OC | 1 | No SP detected |
| gi|118470278 | MSMEG_4932 | YP_889184.1 | murA | Rv1315 | UDP-N-acetylglucosamine 1-carboxyvinyltransferase [Mycobacterium smegmatis str. MC2 155] | 4.75 | 12009.65 | COG0766M | 1 | No SP detected |
| gi|118470303 | MSMEG_1951 | YP_886317.1 | - | * | hypothetical protein MSMEG_1951 [Mycobacterium smegmatis str. MC2 155] | 6.7 | 24747.81 | - | 0 | No SP detected |
| gi|118470328 | MSMEG_3859 | YP_888150.1 | ppm1 | Rv2051c | glycosyl transferase, group 2 family protein [Mycobacterium smegmatis str. MC2 155] | 6.39 | 29106.05 | COG0463M | 1 | No SP detected |
| gi|118470335 | MSMEG_0643 | YP_885053.1 |  | * | extracellular solute-binding protein, family protein 5, putative [Mycobacterium smegmatis str. MC2 155] | 4.53 | 60560.38 | COG0747E | 1 | SP detected |
| gi|118470343 | MSMEG_5175 | YP_889421.1 | - | Rv1151c | NAD-dependent deacetylase [Mycobacterium smegmatis str. MC2 155] | 4.69 | 26126.43 | COG0846K | 1 | No SP detected |
| gi|118470346 | MSMEG_3148 | YP_887463.1 | - | Rv1480 | hypothetical protein MSMEG_3148 [Mycobacterium smegmatis str. MC2 155] | 7.09 | 34613.47 | COG1721R | 0 | No SP detected |
| gi|118470373 | MSMEG_2405 | YP_886745.1 | - | * | MarR-family protein transcriptional regulator [Mycobacterium smegmatis str. MC2 155] | 6.05 | 18411.86 | COG0454KR | 0 | No SP detected |
| gi|118470404 | MSMEG_3251 | YP_887562.1 | cysA1 | Rv2397c | branched-chain amino acid ABC transporter ATP-binding protein [Mycobacterium smegmatis str. MC2 155] | 9.45 | 27198.35 | COG0410E | 0 | No SP detected |
| gi|118470425 | MSMEG_0913 | YP_885316.1 | umaA | Rv0469 | methoxy mycolic acid synthase 1 [Mycobacterium smegmatis str. MC2 155] | 4.96 | 33068.69 | COG2230M | 0 | No SP detected |
| gi|118470459 | MSMEG_6077 | YP_890299.1 | - | Rv3583c | transcriptional regulator, CarD family protein [Mycobacterium smegmatis str. MC2 155] | 5.49 | 17960.47 | COG1329K | 0 | No SP detected |
| gi|118470488 | MSMEG_0940 | YP_885343.1 | - | Rv0497 | hypothetical protein MSMEG_0940 [Mycobacterium smegmatis str. MC2 155] | 4.21 | 38015.63 | - | 3 | No SP detected |
| gi|118470497 | MSMEG_5893 | YP_890119.1 | - | Rv3492c | hypothetical protein MSMEG_5893 [Mycobacterium smegmatis str. MC2 155] | 9.59 | 18099.93 | - | 1 | SP detected |
| gi|118470498 | MSMEG_2762 | YP_887092.1 | suhB | Rv2701c | inositol-1-monophosphatase [Mycobacterium smegmatis str. MC2 155] | 4.85 | 29226.88 | COG0483G | 4 | No SP detected |
| gi|118470516 | MSMEG_2698 | YP_887033.1 | - | Rv2740 | hypothetical protein MSMEG_2698 [Mycobacterium smegmatis str. MC2 155] | 7.9 | 15414.76 | COG4308Q | 0 | No SP detected |
| gi|118470532 | MSMEG_2261 | YP_886613.1 | - | * | hypothetical protein MSMEG_2261 [Mycobacterium smegmatis str. MC2 155] | 5.13 | 19386.07 | COG2427S | 0 | No SP detected |
| gi|118470564 | MSMEG_3247 | YP_887558.1 |  | * | branched-chain amino acid ABC transporter substrate-binding protein [Mycobacterium smegmatis str. MC2 155] | 4.35 | 40502.13 | COG0683E | 0 | No SP detected |
| gi|118470567 | MSMEG_0005 | YP_884428.1 | gyrB | Rv0005 | DNA gyrase subunit B [Mycobacterium smegmatis str. MC2 155] | 5.8 | 74541.21 | COG0187L | 0 | No SP detected |
| gi|118470577 | MSMEG_4323 | YP_888598.1 | aceE | Rv2241 | pyruvate dehydrogenase subunit E1 [Mycobacterium smegmatis str. MC2 155] | 5.41 | 103076.54 | COG2609C | 1 | No SP detected |
| gi|118470599 | MSMEG_4293 | YP_888570.1 | glnE | Rv2221c | glutamate-ammonia-ligase adenylyltransferase [Mycobacterium smegmatis str. MC2 155] | 5.76 | 109399.77 | COG1391OT | 0 | No SP detected |
| gi|118470606 | MSMEG_2373 | YP_886715.1 | ilvH | Rv3002c | acetolactate synthase 3 regulatory subunit [Mycobacterium smegmatis str. MC2 155] | 6.63 | 18395.14 | COG0440E | 0 | No SP detected |
| gi|118470622 | MSMEG_5368 | YP_889611.1 | ehuB | * | ectoine/hydroxyectoine ABC transporter solute-binding protein [Mycobacterium smegmatis str. MC2 155] | 4.8 | 33063.14 | COG0834ET | 1 | SP detected |
| gi|118470627 | MSMEG_4938 | YP_889190.1 | atpA | Rv1308 | F0F1 ATP synthase subunit alpha [Mycobacterium smegmatis str. MC2 155] | 4.84 | 58888.7 | COG0056C | 0 | No SP detected |
| gi|118470637 | MSMEG_4217 | YP_888494.1 | wag31 | Rv2145c | DivIVA protein [Mycobacterium smegmatis str. MC2 155] | 4.67 | 29544.39 | COG3599D | 0 | No SP detected |
| gi|118470667 | MSMEG_5121 | YP_889368.1 | - | Rv1178 | N-succinyldiaminopimelate aminotransferase [Mycobacterium smegmatis str. MC2 155] | 5.54 | 39371.89 | COG0436E | 0 | No SP detected |
| gi|118470668 | MSMEG_3080 | YP_887396.1 | - | Rv1422 | hypothetical protein MSMEG_3080 [Mycobacterium smegmatis str. MC2 155] | 6.26 | 35823.22 | COG0391S | 0 | No SP detected |
| gi|118470677 | MSMEG_5401 | YP_889642.1 | - | * | hypothetical protein MSMEG_5401 [Mycobacterium smegmatis str. MC2 155] | 5.83 | 17626.11 | - | 0 | No SP detected |
| gi|118470681 | MSMEG_4710 | YP_888970.1 | pdhC | Rv2495c | branched-chain alpha-keto acid dehydrogenase subunit E2 [Mycobacterium smegmatis str. MC2 155] | 5.3 | 42841.76 | COG0508C | 0 | No SP detected |
| gi|118470684 | MSMEG_4349 | YP_888624.1 |  | Rv2264c | conserved hypothetical proline rich protein [Mycobacterium smegmatis str. MC2 155] | 4.49 | 60081.4 | COG0443O | 0 | No SP detected |
| gi|118470690 | MSMEG_0244 | YP_884659.1 | prrA | Rv0903c | two component response transcriptional regulatory protein prra [Mycobacterium smegmatis str. MC2 155] | 5.11 | 26981.71 | COG0745TK | 0 | No SP detected |
| gi|118470708 | MSMEG_0736 | YP_885142.1 | - | Rv0383c | hypothetical protein MSMEG_0736 [Mycobacterium smegmatis str. MC2 155] | 10.61 | 29040.41 | - | 1 | No SP detected |
| gi|118470722 | MSMEG_3079 | YP_887395.1 | - | Rv1421 | hypothetical protein MSMEG_3079 [Mycobacterium smegmatis str. MC2 155] | 5.34 | 31942.31 | COG1660R | 0 | No SP detected |
| gi|118470740 | MSMEG_1244 | YP_885635.1 | - | * | hypothetical protein MSMEG_1244 [Mycobacterium smegmatis str. MC2 155] | 5.57 | 126164.59 | COG1122P | 0 | No SP detected |
| gi|118470742 | MSMEG_0007 | YP_884430.1 | - | Rv0007 | hypothetical protein MSMEG_0007 [Mycobacterium smegmatis str. MC2 155] | 8.11 | 27228.65 | - | 2 | No SP detected |
| gi|118470792 | MSMEG_1986 | YP_886350.1 | leuB | Rv2995c | tartrate dehydrogenase [Mycobacterium smegmatis str. MC2 155] | 4.84 | 39864.23 | COG0473CE | 0 | No SP detected |
| gi|118470805 | MSMEG_4699 | YP_888960.1 | gdh | Rv2476c | NAD-glutamate dehydrogenase [Mycobacterium smegmatis str. MC2 155] | 5.18 | 174095.38 | COG2902E | 0 | No SP detected |
| gi|118470826 | MSMEG_6612 | YP_890823.1 | moxR1 | Rv1479 | ATPase, MoxR family protein [Mycobacterium smegmatis str. MC2 155] | 5.71 | 36308.61 | COG0714R | 0 | No SP detected |
| gi|118470874 | MSMEG_0464 | YP_884878.1 | - | * | phosphomethylpyrimidine kinase [Mycobacterium smegmatis str. MC2 155] | 4.81 | 27975.73 | COG0351H | 0 | No SP detected |
| gi|118470901 | MSMEG_4254 | YP_888530.1 | fadD15 | Rv2187 | AMP-binding enzyme [Mycobacterium smegmatis str. MC2 155] | 5.67 | 70086.73 | COG1020Q, COG1022I, COG3320Q | 3 | No SP detected |
| gi|118470922 | MSMEG_0222 | YP_884637.1 | - | Rv0184 | hypothetical protein MSMEG_0222 [Mycobacterium smegmatis str. MC2 155] | 9.7 | 26650.05 | - | 0 | No SP detected |
| gi|118470980 | MSMEG_6127 | YP_890348.1 | rsfB | Rv3687c | anti-anti-sigma factor [Mycobacterium smegmatis str. MC2 155] | 4.33 | 12028.82 | COG1366T | 0 | No SP detected |
| gi|118470988 | MSMEG_0703 | YP_885110.1 | - | Rv1929c | hypothetical protein MSMEG_0703 [Mycobacterium smegmatis str. MC2 155] | 5.97 | 22432.8 | - | 0 | No SP detected |
| gi|118470992 | MSMEG_0100 | YP_884517.1 | ptbB | Rv0153c | phosphotyrosine protein phosphatase ptpb [Mycobacterium smegmatis str. MC2 155] | 4.8 | 29272.61 | COG2365T | 0 | No SP detected |
| gi|118471026 | MSMEG_5471 | YP_889709.1 | galU | Rv0993 | UTP-glucose-1-phosphate uridylyltransferase [Mycobacterium smegmatis str. MC2 155] | 5.28 | 31685.51 | COG1210M | 0 | No SP detected |
| gi|118471030 | MSMEG_1826 | YP_886197.1 | wbbL1 | Rv3265c | dTDP-RhA:a-D-GlcNAc-diphosphoryl polyprenol, a-3-L-rhamnosyl [Mycobacterium smegmatis str. MC2 155] | 9.01 | 32695.79 | COG1216R | 0 | No SP detected |
| gi|118471041 | MSMEG_1470 | YP_885852.1 | rplF | Rv0719 | 50S ribosomal protein L6 [Mycobacterium smegmatis str. MC2 155] | 9.99 | 19453.35 | COG0097J | 0 | No SP detected |
| gi|118471055 | MSMEG_4283 | YP_888560.1 | dlaT | Rv2215 | dihydrolipoamide acetyltransferase [Mycobacterium smegmatis str. MC2 155] | 4.65 | 61029.52 | COG0508C | 0 | No SP detected |
| gi|118471065 | MSMEG_1713 | YP_886089.1 | araB | * | ribulokinase [Mycobacterium smegmatis str. MC2 155] | 5.26 | 60432.44 | COG1069C | 0 | No SP detected |
| gi|118471088 | MSMEG_1401 | YP_885786.1 | tuf | Rv0685 | elongation factor Tu [Mycobacterium smegmatis str. MC2 155] | 5.18 | 43735.72 | COG0050J | 0 | No SP detected |
| gi|118471090 | MSMEG_1638 | YP_886015.1 | - | Rv3364c | Roadblock/LC7 domain protein [Mycobacterium smegmatis str. MC2 155] | 4.84 | 14168.15 | COG2018R | 0 | No SP detected |
| gi|118471121 | MSMEG_2285 | YP_886635.1 | - | Rv1405c | methyltransferase type 11 [Mycobacterium smegmatis str. MC2 155] | 4.77 | 28806.6 | COG0500QR | 1 | No SP detected |
| gi|118471133 | MSMEG_4940 | YP_889192.1 | atpF | Rv1306 | F0F1 ATP synthase subunit B [Mycobacterium smegmatis str. MC2 155] | 4.8 | 17620.78 | COG0711C | 1 | No SP detected |
| gi|118471139 | MSMEG_6043 | YP_890267.1 | otsB | * | trehalose-phosphatase [Mycobacterium smegmatis str. MC2 155] | 4.89 | 25933.08 | COG1877G | 0 | No SP detected |
| gi|118471164 | MSMEG_4644 | YP_888907.1 | mobA | Rv2453c | molybdopterin-guanine dinucleotide biosynthesis protein A [Mycobacterium smegmatis str. MC2 155] | 5.03 | 19650.73 | COG0746H | 0 | No SP detected |
| gi|118471166 | MSMEG_4692 | YP_888953.1 | - | Rv2468c | hypothetical protein MSMEG_4692 [Mycobacterium smegmatis str. MC2 155] | 4.64 | 15899.06 | - | 0 | No SP detected |
| gi|118471195 | MSMEG_4504 | YP_888776.1 | dnaJ2 | Rv2373c | chaperone protein DnaJ [Mycobacterium smegmatis str. MC2 155] | 5.91 | 40335.35 | COG0484O | 0 | No SP detected |
| gi|118471197 | MSMEG_4954 | YP_889206.1 | rho | Rv1297 | transcription termination factor Rho [Mycobacterium smegmatis str. MC2 155] | 6.71 | 71747.63 | COG1158K | 0 | No SP detected |
| gi|118471214 | MSMEG_5694 | YP_889927.1 | - | Rv0875c | hypothetical protein MSMEG_5694 [Mycobacterium smegmatis str. MC2 155] | 6.9 | 17861.45 | - | 1 | SP detected |
| gi|118471232 | MSMEG_3418 | YP_887722.1 | - | Rv0910 | hypothetical protein MSMEG_3418 [Mycobacterium smegmatis str. MC2 155] | 7.95 | 15480.9 | - | 1 | No SP detected |
| gi|118471239 | MSMEG_2378 | YP_886720.1 | serA | Rv2996c | D-3-phosphoglycerate dehydrogenase [Mycobacterium smegmatis str. MC2 155] | 4.9 | 54357.96 | COG0111HE | 0 | No SP detected |
| gi|118471255 | MSMEG_0934 | YP_885337.1 | - | Rv0487 | hypothetical protein MSMEG_0934 [Mycobacterium smegmatis str. MC2 155] | 5.99 | 19013.68 | - | 0 | No SP detected |
| gi|118471271 | MSMEG_3216 | YP_887530.1 | - | Rv1608c | peroxiredoxin Q [Mycobacterium smegmatis str. MC2 155] | 6.28 | 16555.95 | COG1225O | 0 | No SP detected |
| gi|118471281 | MSMEG_2656 | YP_886993.1 | gpsI | Rv2783c | polynucleotide phosphorylase/polyadenylase [Mycobacterium smegmatis str. MC2 155] | 4.76 | 81027.13 | COG1185J | 0 | No SP detected |
| gi|118471293 | MSMEG_2648 | YP_886985.1 | - | Rv2794c | Sfp-type phosphopantetheinyl transferase [Mycobacterium smegmatis str. MC2 155] | 5.13 | 23949.29 | COG2977Q | 0 | No SP detected |
| gi|118471300 | MSMEG_0641 | YP_885051.1 | dppB | Rv3665c | binding-protein-dependent transport systems inner membrane component [Mycobacterium smegmatis str. MC2 155] | 9.51 | 35110.58 | COG0601EP | 6 | No SP detected |
| gi|118471308 | MSMEG_1342 | YP_885730.1 | - | Rv0637 | (3R)-hydroxyacyl-ACP dehydratase subunit HadC [Mycobacterium smegmatis str. MC2 155] | 5.18 | 18929.68 | COG2030I | 0 | No SP detected |
| gi|118471312 | MSMEG_6150 | YP_890371.1 |  | * | 4-carboxymuconolactone decarboxylase [Mycobacterium smegmatis str. MC2 155] | 5.37 | 13061.85 | COG0599S | 0 | No SP detected |
| gi|118471316 | MSMEG_2026 | YP_886388.1 | - | * | short chain dehydrogenase [Mycobacterium smegmatis str. MC2 155] | 5.18 | 28711.17 | COG1028IQR | 1 | No SP detected |
| gi|118471432 | MSMEG_1966 | YP_886331.1 | - | * | hypothetical protein MSMEG_1966 [Mycobacterium smegmatis str. MC2 155] | 4.37 | 14418.46 | - | 0 | No SP detected |
| gi|118471450 | MSMEG_1556 | YP_885936.1 | rplM | Rv3443c | 50S ribosomal protein L13 [Mycobacterium smegmatis str. MC2 155] | 9.95 | 16118.66 | COG0102J | 0 | No SP detected |
| gi|118471467 | MSMEG_0976 | YP_885378.1 | - | * | hypothetical protein MSMEG_0976 [Mycobacterium smegmatis str. MC2 155] | 9.6 | 5978.96 | - | 1 | No SP detected |
| gi|118471488 | MSMEG_1061 | YP_885457.1 | - | * | phosphohydrolase [Mycobacterium smegmatis str. MC2 155] | 4.93 | 22734.8 | COG3576R | 0 | No SP detected |
| gi|118471511 | MSMEG_2490 | YP_886829.1 | - | * | decarboxylase [Mycobacterium smegmatis str. MC2 155] | 4.67 | 21559.56 | COG3473Q | 0 | No SP detected |
| gi|118471527 | MSMEG_4326 | YP_888601.1 | acpP | Rv2244 | acyl carrier protein [Mycobacterium smegmatis str. MC2 155] | 3.92 | 10578.8 | COG0236IQ | 0 | No SP detected |
| gi|118471545 | MSMEG_4308 | YP_888585.1 |  | Rv2232 | 5'-nucleotidase [Mycobacterium smegmatis str. MC2 155] | 4.83 | 24195.24 | COG0546R | 0 | No SP detected |
| gi|118471547 | MSMEG_5512 | YP_889749.1 | - | Rv0958 | magnesium chelatase [Mycobacterium smegmatis str. MC2 155] | 5.05 | 50033.82 | COG1239H | 0 | No SP detected |
| gi|118471592 | MSMEG_4563 | YP_888833.1 | - | Rv0133 | puromycin N-acetyltransferase [Mycobacterium smegmatis str. MC2 155] | 9.09 | 22579.84 | COG0454KR | 0 | No SP detected |
| gi|118471597 | MSMEG_1076 | YP_885472.1 | - | * | hypothetical protein MSMEG_1076 [Mycobacterium smegmatis str. MC2 155] | 4.96 | 9549.86 | - | 1 | SP detected |
| gi|118471598 | MSMEG_2363 | YP_886706.1 |  | Rv3013 | amino acid-binding ACT [Mycobacterium smegmatis str. MC2 155] | 4.97 | 23037.29 | COG0460E | 0 | No SP detected |
| gi|118471625 | MSMEG_5639 | YP_889873.1 | echA6 | Rv0905 | enoyl-CoA hydratase [Mycobacterium smegmatis str. MC2 155] | 5.22 | 26054.85 | COG1024I | 1 | No SP detected |
| gi|118471632 | MSMEG_2961 | YP_887280.1 | secD | Rv2587c | preprotein translocase subunit SecD [Mycobacterium smegmatis str. MC2 155] | 5.02 | 63646.71 | COG0342U | 5 | No SP detected |
| gi|118471642 | MSMEG_0690 | YP_885098.1 | - | Rv0338c | iron-sulfur cluster-binding protein [Mycobacterium smegmatis str. MC2 155] | 6.4 | 110905.36 | COG0247C | 7 | No SP detected |
| gi|118471646 | MSMEG_0408 | YP_884821.1 | pks7 | Rv1661 | type I modular polyketide synthase [Mycobacterium smegmatis str. MC2 155] | 5.01 | 389863.67 | COG3321Q | 0 | No SP detected |
| gi|118471661 | MSMEG_4923 | YP_889178.1 | - | Rv1321 | hypothetical protein MSMEG_4923 [Mycobacterium smegmatis str. MC2 155] | 5.56 | 25093.73 | COG1637L | 0 | No SP detected |
| gi|118471695 | MSMEG_6616 | YP_890827.1 | - | Rv1895 | S-(hydroxymethyl)glutathione dehydrogenase [Mycobacterium smegmatis str. MC2 155] | 4.96 | 42463.56 | COG1063ER | 0 | No SP detected |
| gi|118471704 | MSMEG_3461 | YP_887765.1 | katG | Rv1908c | catalase/peroxidase HPI [Mycobacterium smegmatis str. MC2 155] | 4.99 | 81998.46 | COG0376P | 0 | No SP detected |
| gi|118471714 | MSMEG_4674 | YP_888937.1 | tig | Rv2462c | trigger factor [Mycobacterium smegmatis str. MC2 155] | 4.3 | 51655.34 | COG0544O | 0 | No SP detected |
| gi|118471722 | MSMEG_0372 | YP_884785.1 | fabG | Rv0242c | 3-ketoacyl-(acyl-carrier-protein) reductase [Mycobacterium smegmatis str. MC2 155] | 6.04 | 46830.21 | COG1028IQR | 0 | No SP detected |
| gi|118471723 | MSMEG_2408 | YP_886748.1 |  | Rv2971 | 2,5-diketo-D-gluconic acid reductase A [Mycobacterium smegmatis str. MC2 155] | 5.06 | 30221 | COG0656R | 0 | No SP detected |
| gi|118471734 | MSMEG_0048 | YP_884466.1 | - | Rv2991 | pyridoxamine 5'-phosphate oxidase family protein [Mycobacterium smegmatis str. MC2 155] | 5.65 | 18885.52 | COG0748P | 0 | No SP detected |
| gi|118471755 | MSMEG_4976 | YP_889227.1 |  | * | isochorismatase hydrolase [Mycobacterium smegmatis str. MC2 155] | 4.6 | 20699.54 | COG1335Q | 0 | No SP detected |
| gi|118471759 | MSMEG_4121 | YP_888403.1 | - | * | GntR-family protein transcriptional regulator [Mycobacterium smegmatis str. MC2 155] | 6.6 | 26836.43 | COG2186K | 0 | No SP detected |
| gi|118471776 | MSMEG_2443 | YP_886783.1 | - | Rv2901c | hypothetical protein MSMEG_2443 [Mycobacterium smegmatis str. MC2 155] | 4.65 | 12205.85 | - | 0 | No SP detected |
| gi|118471786 | MSMEG_0035 | YP_884455.1 | - | Rv0020c | FHA domain protein [Mycobacterium smegmatis str. MC2 155] | 4.77 | 52025.88 | COG1716T | 0 | No SP detected |
| gi|118471796 | MSMEG_6081 | YP_890303.1 | - | Rv3587c | hypothetical protein MSMEG_6081 [Mycobacterium smegmatis str. MC2 155] | 4.7 | 26181.06 | - | 1 | SP detected |
| gi|118471799 | MSMEG_1353 | YP_885740.1 |  | Rv0647c | ABC1 family protein [Mycobacterium smegmatis str. MC2 155] | 6.49 | 52347.15 | COG0661R | 0 | No SP detected |
| gi|118471823 | MSMEG_1714 | YP_886090.1 | - | * | L-ribulose-5-phosphate 4-epimerase [Mycobacterium smegmatis str. MC2 155] | 6.14 | 15646.64 | COG0235G | 0 | No SP detected |
| gi|118471824 | MSMEG_3641 | YP_887944.1 | - | Rv1836c | hypothetical protein MSMEG_3641 [Mycobacterium smegmatis str. MC2 155] | 4.38 | 80661.19 | COG2304R | 0 | No SP detected |
| gi|118471835 | MSMEG_6919 | YP_891114.1 | mtc28 | Rv0040c | proline-rich 28 kDa antigen [Mycobacterium smegmatis str. MC2 155] | 5.1 | 31728.05 | COG1905C | 1 | SP detected |
| gi|118471837 | MSMEG_1881 | YP_886246.1 | secA1 | Rv3240c | preprotein translocase subunit SecA [Mycobacterium smegmatis str. MC2 155] | 5.44 | 107001.78 | COG0653U | 0 | No SP detected |
| gi|118471844 | MSMEG_0028 | YP_884449.1 | pknB | Rv0014c | serine-threonine protein kinase [Mycobacterium smegmatis str. MC2 155] | 5.15 | 66317.79 | COG0515RTKL, COG2815S | 1 | No SP detected |
| gi|118471895 | MSMEG_0632 | YP_885042.1 | - | Rv0307c | hypothetical protein MSMEG_0632 [Mycobacterium smegmatis str. MC2 155] | 8.09 | 17827.47 | - | 0 | No SP detected |
| gi|118471903 | MSMEG_1178 | YP_885570.1 | - | * | transcriptional regulator [Mycobacterium smegmatis str. MC2 155] | 5.38 | 33125.88 | COG1737K | 0 | No SP detected |
| gi|118471962 | MSMEG_5768 | YP_889997.1 | - | Rv0825c | hypothetical protein MSMEG_5768 [Mycobacterium smegmatis str. MC2 155] | 7.64 | 21457.76 | COG1309K | 1 | No SP detected |
| gi|118471980 | MSMEG_1341 | YP_885729.1 | - | Rv0636 | MaoC family protein [Mycobacterium smegmatis str. MC2 155] | 5.3 | 14857.94 | COG2030I | 0 | No SP detected |
| gi|118472022 | MSMEG_0953 | YP_885356.1 | hemC | Rv0510 | porphobilinogen deaminase [Mycobacterium smegmatis str. MC2 155] | 4.65 | 33259.65 | COG0181H | 0 | No SP detected |
| gi|118472053 | MSMEG_1399 | YP_885785.1 | rpsG | Rv0683 | 30S ribosomal protein S7 [Mycobacterium smegmatis str. MC2 155] | 10.56 | 17600.37 | COG0049J | 0 | No SP detected |
| gi|118472062 | MSMEG_1443 | YP_885826.1 | rplP | Rv0708 | 50S ribosomal protein L16 [Mycobacterium smegmatis str. MC2 155] | 10.52 | 15742.19 | COG0197J | 0 | No SP detected |
| gi|118472100 | MSMEG_2728 | YP_887062.1 | glnQ | Rv2564 | glutamate transport ATP-binding protein GluA [Mycobacterium smegmatis str. MC2 155] | 6.6 | 28269.54 | COG1126E | 0 | No SP detected |
| gi|118472105 | MSMEG_4192 | YP_888471.1 | - | Rv2134c | hypothetical protein MSMEG_4192 [Mycobacterium smegmatis str. MC2 155] | 4.62 | 21216.95 | - | 0 | No SP detected |
| gi|118472123 | MSMEG_0098 | YP_884514.1 | - | * | methyltransferase [Mycobacterium smegmatis str. MC2 155] | 8.81 | 25126.11 | COG0500QR | 0 | No SP detected |
| gi|118472151 | MSMEG_2723 | YP_887057.1 | recA | Rv2737c | protein RecA [Mycobacterium smegmatis str. MC2 155] | 5.72 | 32865.51 | COG0468L | 0 | No SP detected |
| gi|118472166 | MSMEG_5244 | YP_889490.1 | devR | Rv3133c | two component transcriptional regulatory protein devr [Mycobacterium smegmatis str. MC2 155] | 5.51 | 22925.56 | COG2197TK | 0 | No SP detected |
| gi|118472168 | MSMEG_3219 | YP_887533.1 | trpC | Rv1611 | indole-3-glycerol-phosphate synthase [Mycobacterium smegmatis str. MC2 155] | 5 | 28323.29 | COG0134E | 0 | No SP detected |
| gi|118472180 | MSMEG_0965 | YP_885367.1 | MspA | - | porin [Mycobacterium smegmatis str. MC2 155] | 4.31 | 19488.66 | COG0580G | 1 | No SP detected |
| gi|118472203 | MSMEG_1414 | YP_885797.1 |  | Rv2323c | Amidinotransferase [Mycobacterium smegmatis str. MC2 155] | 4.88 | 32188.32 | COG1834E | 0 | No SP detected |
| gi|118472240 | MSMEG_0220 | YP_884635.1 | - | Rv0183 | monoglyceride lipase [Mycobacterium smegmatis str. MC2 155] | 6.62 | 29882.18 | COG2267I | 0 | No SP detected |
| gi|118472244 | MSMEG_6163 | YP_890383.1 | - | Rv3651 | hypothetical protein MSMEG_6163 [Mycobacterium smegmatis str. MC2 155] | 5.35 | 36946.92 | - | 0 | No SP detected |
| gi|118472263 | MSMEG_1439 | YP_885822.1 | rplB | Rv0704 | 50S ribosomal protein L2 [Mycobacterium smegmatis str. MC2 155] | 11.39 | 30576.79 | COG0090J | 0 | No SP detected |
| gi|118472300 | MSMEG_4469 | YP_888742.1 | CbiQ | Rv2325c | cobalt transport protein [Mycobacterium smegmatis str. MC2 155] | 11.56 | 30110.69 | COG0619P | 4 | No SP detected |
| gi|118472323 | MSMEG_0059 | YP_884477.1 |  | Rv3868 | ATPase, AAA family protein [Mycobacterium smegmatis str. MC2 155] | 5.02 | 62147.92 | COG0464O | 1 | No SP detected |
| gi|118472339 | MSMEG_2839 | YP_887159.1 | - | * | transcriptional accessory protein [Mycobacterium smegmatis str. MC2 155] | 5.84 | 85418.63 | COG2183K | 0 | No SP detected |
| gi|118472342 | MSMEG_0067 | YP_884485.1 | - | Rv3876 | hypothetical protein MSMEG_0067 [Mycobacterium smegmatis str. MC2 155] | 5.89 | 50938.2 | COG0455D | 0 | No SP detected |
| gi|118472345 | MSMEG_5285 | YP_889531.1 | - | Rv1062 | phospholipase, patatin family protein [Mycobacterium smegmatis str. MC2 155] | 4.43 | 30070.77 | COG1752R | 0 | No SP detected |
| gi|118472363 | MSMEG_4505 | YP_888777.1 | hrcA | Rv2374c | heat-inducible transcription repressor [Mycobacterium smegmatis str. MC2 155] | 5.4 | 36747.76 | COG1420K | 0 | No SP detected |
| gi|118472437 | MSMEG_5225 | YP_889471.1 | - | Rv1109c | hypothetical protein MSMEG_5225 [Mycobacterium smegmatis str. MC2 155] | 4.47 | 21891.27 | - | 0 | No SP detected |
| gi|118472440 | MSMEG_1372 | YP_885758.1 |  | Rv1218c | ABC transporter ATP-binding protein [Mycobacterium smegmatis str. MC2 155] | 6.22 | 26629.64 | COG1129G | 0 | No SP detected |
| gi|118472444 | MSMEG_6761 | YP_890968.1 | glpD2 | Rv3302c | glycerol-3-phosphate dehydrogenase 2 [Mycobacterium smegmatis str. MC2 155] | 6.45 | 60885.8 | COG0578C | 0 | No SP detected |
| gi|118472461 | MSMEG_6757 | YP_890965.1 | - | * | glycerol operon regulatory protein [Mycobacterium smegmatis str. MC2 155] | 6.76 | 26775.43 | COG1414K | 0 | No SP detected |
| gi|118472473 | MSMEG_0250 | YP_884665.1 | mmpL3 | Rv0206c | membrane protein, MmpL family protein [Mycobacterium smegmatis str. MC2 155] | 5.87 | 109399.33 | COG2409R | 12 | No SP detected |
| gi|118472484 | MSMEG_5942 | YP_890169.1 | - | * | AMP-dependent synthetase and ligase [Mycobacterium smegmatis str. MC2 155] | 6.62 | 65755.86 | COG1716T | 10 | No SP detected |
| gi|118472491 | MSMEG_3255 | YP_887566.1 | - | * | DoxX subfamily protein, putative [Mycobacterium smegmatis str. MC2 155] | 9.73 | 30330.39 | COG2259S | 0 | No SP detected |
| gi|118472497 | MSMEG_6779 | YP_890987.1 | - | Rv2036 | hypothetical protein MSMEG_6779 [Mycobacterium smegmatis str. MC2 155] | 10.4 | 23193.67 | - | 0 | No SP detected |
| gi|118472509 | MSMEG_6091 | YP_890312.1 | clpC1 | Rv3596c | negative regulator of genetic competence ClpC/mecB [Mycobacterium smegmatis str. MC2 155] | 5.46 | 93614.47 | COG2256L | 0 | No SP detected |
| gi|118472524 | MSMEG_4561 | YP_888832.1 |  | * | ABC Fe3+-siderophores transporter, periplasmic binding protein [Mycobacterium smegmatis str. MC2 155] | 4.57 | 36463.25 | COG0614P | 0 | SP detected |
| gi|118472550 | MSMEG_0949 | YP_885352.1 | - | Rv0505c | HAD-superfamily protein subfamily protein IB hydrolase, TIGR01490 [Mycobacterium smegmatis str. MC2 155] | 5.8 | 31352.49 | COG0560E | 1 | No SP detected |
| gi|118472552 | MSMEG_5246 | YP_889492.1 | - | Rv2032 | hypothetical protein MSMEG_5246 [Mycobacterium smegmatis str. MC2 155] | 5.19 | 36220.93 | COG0778C | 0 | No SP detected |
| gi|118472583 | MSMEG_4437 | YP_888712.1 | ctaD | Rv3043c | cytochrome c oxidase, subunit I [Mycobacterium smegmatis str. MC2 155] | 6.48 | 63672.73 | COG0843C | 12 | No SP detected |
| gi|118472587 | MSMEG_2939 | YP_887258.1 | - | Rv2604c | glutamine amidotransferase subunit PdxT [Mycobacterium smegmatis str. MC2 155] | 5.3 | 20724.79 | COG0311H | 0 | No SP detected |
| gi|118472590 | MSMEG_4183 | YP_888462.1 | - | * | phosphoglycolate phosphatase, chromosomal [Mycobacterium smegmatis str. MC2 155] | 4.55 | 24111.47 | COG0637R | 0 | No SP detected |
| gi|118472638 | MSMEG_2090 | YP_886450.1 | ftsX | Rv3101c | putative cell division protein FtsX [Mycobacterium smegmatis str. MC2 155] | 8.85 | 32712.16 | COG2177D | 4 | No SP detected |
| gi|118472640 | MSMEG_3479 | YP_887783.1 | tpx | Rv1932 | thiol peroxidase [Mycobacterium smegmatis str. MC2 155] | 4.39 | 16831.99 | COG2077O | 0 | No SP detected |
| gi|118472646 | MSMEG_3078 | YP_887394.1 | uvrC | Rv1420 | excinuclease ABC subunit C [Mycobacterium smegmatis str. MC2 155] | 6.08 | 77443.07 | COG0322L | 1 | No SP detected |
| gi|118472721 | MSMEG_1834 | YP_886204.1 | manB | Rv3257c | phosphomannomutase/phosphoglucomutase [Mycobacterium smegmatis str. MC2 155] | 4.73 | 49504.52 | COG1109G | 1 | No SP detected |
| gi|118472773 | MSMEG_1611 | YP_885989.1 | - | * | transcriptional regulator, TetR family protein, putative [Mycobacterium smegmatis str. MC2 155] | 5.86 | 25238.82 | COG1309K | 0 | No SP detected |
| gi|118472775 | MSMEG_5873 | YP_890100.1 | - | Rv0756c | hypothetical protein MSMEG_5873 [Mycobacterium smegmatis str. MC2 155] | 4.5 | 28353.3 | - | 0 | No SP detected |
| gi|118472782 | MSMEG_6596 | YP_890808.1 | - | Rv2172c | hypothetical protein MSMEG_6596 [Mycobacterium smegmatis str. MC2 155] | 4.76 | 33311.1 | COG1180O | 0 | No SP detected |
| gi|118472813 | MSMEG_3833 | YP_888124.1 | rpsA | Rv1630 | 30S ribosomal protein S1 [Mycobacterium smegmatis str. MC2 155] | 4.77 | 53315.85 | COG0539J | 0 | No SP detected |
| gi|118472837 | MSMEG_0415 | YP_884828.1 | - | Rv0245 | NADH-fmn oxidoreductase [Mycobacterium smegmatis str. MC2 155] | 5.23 | 16892.18 | COG1853R | 0 | No SP detected |
| gi|118472873 | MSMEG_0057 | YP_884475.1 | - | Rv3866 | hypothetical protein MSMEG_0057 [Mycobacterium smegmatis str. MC2 155] | 5.04 | 30581.74 | - | 0 | No SP detected |
| gi|118472876 | MSMEG_1707 | YP_886083.1 | - | * | phosphatase YfbT [Mycobacterium smegmatis str. MC2 155] | 4.65 | 22798.71 | COG0637R | 0 | No SP detected |
| gi|118472891 | MSMEG_4261 | YP_888538.1 | qcrC | Rv2194 | ubiquinol-cytochrome c reductase cytochrome c subunit [Mycobacterium smegmatis str. MC2 155] | 7.69 | 27846.57 | COG2010C | 2 | SP detected |
| gi|118472900 | MSMEG_1467 | YP_885849.1 | rplE | Rv0716 | 50S ribosomal protein L5 [Mycobacterium smegmatis str. MC2 155] | 9.68 | 21122.31 | COG0094J | 0 | No SP detected |
| gi|118472918 | MSMEG_0030 | YP_884450.1 | pknA | Rv0015c | serine/threonine protein kinase PknA [Mycobacterium smegmatis str. MC2 155] | 5.53 | 48089.1 | COG0515RTKL | 1 | No SP detected |
| gi|118472922 | MSMEG_0640 | YP_885050.1 | oppD | Rv1281c | oligopeptide transport ATP-binding protein OppD [Mycobacterium smegmatis str. MC2 155] | 5.05 | 36058.01 | COG0444EP | 0 | No SP detected |
| gi|118472965 | MSMEG_6337 | YP_890554.1 | fadE36 | Rv3761c | phosphotransferase enzyme family protein [Mycobacterium smegmatis str. MC2 155] | 5.16 | 38541 | COG1960I | 0 | No SP detected |
| gi|118472988 | MSMEG_2666 | YP_887003.1 | - | Rv2771c | multimeric flavodoxin WrbA [Mycobacterium smegmatis str. MC2 155] | 4.59 | 15885.97 | COG0655R | 0 | No SP detected |
| gi|118472999 | MSMEG_4626 | YP_888890.1 | rne | Rv2444c | ribonuclease, Rne/Rng family protein [Mycobacterium smegmatis str. MC2 155] | 4.54 | 102171.79 | COG0532J, COG1530J | 0 | No SP detected |
| gi|118473019 | MSMEG_5488 | YP_889726.1 | mprA | Rv0981 | DNA-binding response regulator [Mycobacterium smegmatis str. MC2 155] | 5.06 | 25774.41 | COG0745TK | 0 | No SP detected |
| gi|118473034 | MSMEG_4705 | YP_888966.1 | - | Rv2484c | acyltransferase, ws/dgat/mgat subfamily protein [Mycobacterium smegmatis str. MC2 155] | 5.42 | 51879.3 | - | 2 | No SP detected |
| gi|118473049 | MSMEG_5048 | YP_889298.1 | - | Rv1249c | hypothetical protein MSMEG_5048 [Mycobacterium smegmatis str. MC2 155] | 10.51 | 26261.49 | - | 3 | No SP detected |
| gi|118473053 | MSMEG_0954 | YP_885357.1 | hemD | Rv0511 | uroporphyrinogen-III synthase [Mycobacterium smegmatis str. MC2 155] | 5.7 | 63821.4 | COG0007H, COG1587H | 0 | SP detected |
| gi|118473057 | MSMEG_5431 | YP_889670.1 | rplY | Rv1015c | 50S ribosomal protein L25/general stress protein Ctc [Mycobacterium smegmatis str. MC2 155] | 4.56 | 22550.17 | COG1825J | 0 | No SP detected |
| gi|118473062 | MSMEG_3028 | YP_887345.1 | aroE | Rv2552c | shikimate 5-dehydrogenase [Mycobacterium smegmatis str. MC2 155] | 5.33 | 27208.09 | COG0169E | 0 | No SP detected |
| gi|118473066 | MSMEG_4313 | YP_888589.1 | - | * | glyoxalase/bleomycin resistance protein/dioxygenase [Mycobacterium smegmatis str. MC2 155] | 4.86 | 14925.57 | COG0346E | 0 | No SP detected |
| gi|118473092 | MSMEG_6409 | YP_890622.1 | - | Rv3816c | acyltransferase family protein [Mycobacterium smegmatis str. MC2 155] | 10.03 | 28453.9 | COG1835I | 1 | No SP detected |
| gi|118473106 | MSMEG_6105 | YP_890326.1 | ftsH | Rv3610c | cell division protein [Mycobacterium smegmatis str. MC2 155] | 5.77 | 83538.38 | COG0465O | 2 | No SP detected |
| gi|118473118 | MSMEG_4242 | YP_888519.1 | - | Rv2175c | transcriptional regulatory protein [Mycobacterium smegmatis str. MC2 155] | 5.9 | 14803.89 | COG1358J | 0 | No SP detected |
| gi|118473128 | MSMEG_1364 | YP_885750.1 | rplJ | Rv0651 | 50S ribosomal protein L10 [Mycobacterium smegmatis str. MC2 155] | 9 | 18010.75 | COG0244J | 0 | No SP detected |
| gi|118473160 | MSMEG_1442 | YP_885825.1 | rpsC | Rv0707 | 30S ribosomal protein S3 [Mycobacterium smegmatis str. MC2 155] | 10.2 | 30139.14 | COG0092J | 0 | No SP detected |
| gi|118473163 | MSMEG_0441 | YP_884854.1 | - | * | hypothetical protein MSMEG_0441 [Mycobacterium smegmatis str. MC2 155] | 4.5 | 26511.73 | - | 0 | No SP detected |
| gi|118473190 | MSMEG_1436 | YP_885819.1 | rplC | Rv0701 | 50S ribosomal protein L3 [Mycobacterium smegmatis str. MC2 155] | 10.3 | 22863.18 | COG0087J | 0 | No SP detected |
| gi|118473224 | MSMEG_5676 | YP_889909.1 | citA | Rv0889c | citrate synthase 2 [Mycobacterium smegmatis str. MC2 155] | 5.17 | 40332.97 | COG0372C | 0 | No SP detected |
| gi|118473234 | MSMEG_4724 | YP_888984.1 | orn | Rv2511 | oligoribonuclease [Mycobacterium smegmatis str. MC2 155] | 4.96 | 24101.45 | COG1949A | 0 | No SP detected |
| gi|118473235 | MSMEG_4647 | YP_888910.1 |  | * | carbohydrate kinase, PfkB, putative [Mycobacterium smegmatis str. MC2 155] | 4.83 | 28937.75 | COG0524G | 1 | No SP detected |
| gi|118473240 | MSMEG_1469 | YP_885851.1 | rpsH | Rv0718 | 30S ribosomal protein S8 [Mycobacterium smegmatis str. MC2 155] | 9.52 | 14141.3 | COG0096J | 0 | No SP detected |
| gi|118473251 | MSMEG_1711 | YP_886087.1 |  | Rv1218c | ATP binding protein of ABC transporter [Mycobacterium smegmatis str. MC2 155] | 6.15 | 54414.47 | COG1129G | 0 | No SP detected |
| gi|118473255 | MSMEG_4937 | YP_889189.1 | atpG | Rv1309 | F0F1 ATP synthase subunit gamma [Mycobacterium smegmatis str. MC2 155] | 5.71 | 33397.85 | COG0224C | 0 | No SP detected |
| gi|118473274 | MSMEG_6247 | YP_890466.1 | - | Rv3701c | hypothetical protein MSMEG_6247 [Mycobacterium smegmatis str. MC2 155] | 4.76 | 35002.59 | COG4301S | 0 | No SP detected |
| gi|118473298 | MSMEG_0889 | YP_885292.1 | gabD2 | Rv1731 | succinic semialdehyde dehydrogenase [Mycobacterium smegmatis str. MC2 155] | 6.38 | 54505.52 | COG1012C | 1 | No SP detected |
| gi|118473306 | MSMEG_1640 | YP_886017.1 |  | Rv3362c | ATP/GTP-binding protein [Mycobacterium smegmatis str. MC2 155] | 6.09 | 20985.15 | COG2229R | 1 | No SP detected |
| gi|118473366 | MSMEG_2424 | YP_886764.1 | ftsY | Rv2921c | signal recognition particle-docking protein FtsY [Mycobacterium smegmatis str. MC2 155] | 5.18 | 44031.25 | COG0532J, COG0552U | 1 | No SP detected |
| gi|118473375 | MSMEG_0317 | YP_884731.1 | - | Rv0227c | hypothetical protein MSMEG_0317 [Mycobacterium smegmatis str. MC2 155] | 4.53 | 43259.48 | - | 1 | SP detected |
| gi|118473377 | MSMEG_1248 | YP_885639.1 | - | * | hypothetical protein MSMEG_1248 [Mycobacterium smegmatis str. MC2 155] | 5.32 | 25209.42 | - | 0 | No SP detected |
| gi|118473383 | MSMEG_1285 | YP_885675.1 | - | Rv0613c | tetratricopeptide repeat family protein [Mycobacterium smegmatis str. MC2 155] | 4.5 | 88001.32 | COG0653U | 0 | No SP detected |
| gi|118473389 | MSMEG_1808 | YP_886180.1 | sufE | Rv3284 | Fe-S metabolism associated SufE [Mycobacterium smegmatis str. MC2 155] | 4.43 | 15139.34 | COG2166R | 0 | No SP detected |
| gi|118473408 | MSMEG_6104 | YP_890325.1 | folE | Rv3609c | GTP cyclohydrolase I [Mycobacterium smegmatis str. MC2 155] | 8.59 | 22494.88 | COG0302H | 0 | No SP detected |
| gi|118473457 | MSMEG_1435 | YP_885818.1 | rpsM | Rv0700 | 30S ribosomal protein S10 [Mycobacterium smegmatis str. MC2 155] | 9.41 | 11431.31 | COG0051J | 0 | No SP detected |
| gi|118473461 | MSMEG_6434 | YP_890647.1 | - | Rv3850 | hypothetical protein MSMEG_6434 [Mycobacterium smegmatis str. MC2 155] | 11.45 | 23717.58 | - | 0 | No SP detected |
| gi|118473494 | MSMEG_2786 | YP_887117.1 | - | Rv2672 | hydrolase, alpha/beta fold family protein [Mycobacterium smegmatis str. MC2 155] | 4.5 | 54607.4 | COG0596R | 1 | SP detected |
| gi|118473523 | MSMEG_3021 | YP_887339.1 |  | Rv2559c | recombination factor protein RarA [Mycobacterium smegmatis str. MC2 155] | 5.84 | 49478.02 | COG2256L | 0 | No SP detected |
| gi|118473523 | MSMEG_3021 | YP_887339.1 | - | Rv2559c | recombination factor protein RarA [Mycobacterium smegmatis str. MC2 155] | 6.29 | 50547.52 | COG2220R | 0 | No SP detected |
| gi|118473536 | MSMEG_3119 | YP_887434.1 | - | Rv1458c | ABC transporter, ATP-binding subunit [Mycobacterium smegmatis str. MC2 155] | 5.42 | 30625.95 | COG1131V | 0 | No SP detected |
| gi|118473566 | MSMEG_3673 | YP_887976.1 | malQ | Rv1781c | 4-alpha-glucanotransferase [Mycobacterium smegmatis str. MC2 155] | 5.78 | 78022.01 | COG3387G | 0 | No SP detected |
| gi|118473568 | MSMEG_0835 | YP_885239.1 | sodC | Rv0432 | copper/zinc superoxide dismutase [Mycobacterium smegmatis str. MC2 155] | 4.85 | 23213.46 | COG2032P | 0 | SP detected |
| gi|118473659 | MSMEG_4489 | YP_888761.1 | - | Rv2360c | hypothetical protein MSMEG_4489 [Mycobacterium smegmatis str. MC2 155] | 5.41 | 16279.88 | - | 0 | No SP detected |
| gi|118473675 | MSMEG_0977 | YP_885379.1 | - | * | hypothetical protein MSMEG_0977 [Mycobacterium smegmatis str. MC2 155] | 10.13 | 9964.97 | - | 2 | No SP detected |
| gi|118473678 | MSMEG_4672 | YP_888935.1 | clpP2 | Rv2460c | ATP-dependent Clp protease proteolytic subunit [Mycobacterium smegmatis str. MC2 155] | 5.24 | 24066.46 | COG0740OU | 1 | No SP detected |
| gi|118473680 | MSMEG_4320 | YP_888595.1 | ahpE | Rv2238c | alkyl hydroperoxide reductase/ Thiol specific antioxidant/ Mal allergen [Mycobacterium smegmatis str. MC2 155] | 4.96 | 17337.58 | COG1225O | 0 | No SP detected |
| gi|118473685 | MSMEG_0636 | YP_885046.1 | - | Rv0310c | hypothetical protein MSMEG_0636 [Mycobacterium smegmatis str. MC2 155] | 4.82 | 18485.55 | - | 0 | No SP detected |
| gi|118473718 | MSMEG_4697 | YP_888958.1 | - | Rv2474c | hypothetical protein MSMEG_4697 [Mycobacterium smegmatis str. MC2 155] | 5.66 | 23485.62 | - | 0 | No SP detected |
| gi|118473726 | MSMEG_4273 | YP_888550.1 | - | Rv2206 | integral membrane protein [Mycobacterium smegmatis str. MC2 155] | 10.68 | 25652.78 | - | 2 | No SP detected |
| gi|118473730 | MSMEG_3144 | YP_887459.1 | - | Rv1476 | hypothetical protein MSMEG_3144 [Mycobacterium smegmatis str. MC2 155] | 4.37 | 20759.6 | - | 1 | No SP detected |
| gi|118473741 | MSMEG_1712 | YP_886088.1 |  | * | ABC transporter periplasmic-binding protein YtfQ [Mycobacterium smegmatis str. MC2 155] | 4.67 | 34234.5 | COG1879G | 1 | SP detected |
| gi|118473796 | MSMEG_0599 | YP_885010.1 | fadD2 | Rv0270 | acyl-CoA synthetase [Mycobacterium smegmatis str. MC2 155] | 6.57 | 59207.04 | COG0318IQ | 0 | No SP detected |
| gi|118473811 | MSMEG_2089 | YP_886449.1 | ftsE | Rv3102c | cell division ATP-binding protein FtsE [Mycobacterium smegmatis str. MC2 155] | 8.73 | 25455.34 | COG2884D | 0 | No SP detected |
| gi|118473872 | MSMEG_5438 | YP_889677.1 | ksgA | Rv1010 | dimethyladenosine transferase [Mycobacterium smegmatis str. MC2 155] | 7.13 | 32833.44 | COG0030J | 0 | No SP detected |
| gi|118473876 | MSMEG_1340 | YP_885728.1 | - | Rv0635 | hypothetical protein MSMEG_1340 [Mycobacterium smegmatis str. MC2 155] | 4.95 | 17583.7 | - | 0 | No SP detected |
| gi|118473880 | MSMEG_0614 | YP_885025.1 | - | Rv0281 | methyltransferase [Mycobacterium smegmatis str. MC2 155] | 4.65 | 32912.82 | COG3315Q | 0 | No SP detected |
| gi|118473904 | MSMEG_1346 | YP_885733.1 | rplK | Rv0640 | 50S ribosomal protein L11 [Mycobacterium smegmatis str. MC2 155] | 9.35 | 15289.65 | COG0080J | 0 | No SP detected |
| gi|118473908 | MSMEG_6279 | YP_890498.1 | recR | Rv3715c | recombination protein RecR [Mycobacterium smegmatis str. MC2 155] | 4.91 | 22146.35 | COG0353L | 0 | No SP detected |
| gi|118473964 | MSMEG_2690 | YP_887024.1 | ftsK | Rv2748c | DNA translocase FtsK [Mycobacterium smegmatis str. MC2 155] | 5.5 | 99229.37 | COG1674D | 4 | No SP detected |
| gi|118473987 | MSMEG_1521 | YP_885903.1 | rpsJ | Rv3460c | 30S ribosomal protein S13 [Mycobacterium smegmatis str. MC2 155] | 10.78 | 14217.52 | COG0099J | 0 | No SP detected |
| gi|118473994 | MSMEG_3746 | YP_888045.1 | pyrG | Rv1699 | CTP synthetase [Mycobacterium smegmatis str. MC2 155] | 5.41 | 63635.03 | COG0504F | 0 | No SP detected |
| gi|118474040 | MSMEG_6947 | YP_891139.1 | dnaA | Rv0001 | chromosomal replication initiation protein [Mycobacterium smegmatis str. MC2 155] | 9.4 | 55992.88 | COG0593L | 1 | No SP detected |
