# Supplementary material for: Cell wall proteome analysis of Mycobacterium smegmatis strain MC2 155
Source: BMC Microbiol. 2010 Apr 22;10:121. doi: 10.1186/1471-2180-10-121 (PMC2867950; doi:10.1186/1471-2180-10-121)
Supplement: Additional file 2 — Bacterial viable test. A description of bacterial viable test comparison between cells pretreated with trypsin and control. [file 1471-2180-10-121-S2.DOC]

**bacterial viable test**

(1: sample treated with trypsin; 2: control; Cell pellets collected before and after the procedure were resuspended into original volume with Luria Broth medium. Serial dilutions were made and plated out on LB agar plates, and incubated at 37°C for around 15 hours for determining bacterial cell viability.)
