# Supplementary material for: Cell wall proteome analysis of Mycobacterium smegmatis strain MC2 155
Source: BMC Microbiol. 2010 Apr 22;10:121. doi: 10.1186/1471-2180-10-121 (PMC2867950; doi:10.1186/1471-2180-10-121)
Supplement: Additional file 3 — Cell surface-exposed proteins list. A summarization of all the identified cell surface proteins of Mycobacterium smegmatis strain MC2 155. [file 1471-2180-10-121-S3.DOC]

**Additional file 3: Cell surface-exposed proteins list**

| ***ID*** | ***PI*** | ***MW*** | ***Functional category*** | ***Locus*** | ***Proteins name*** |
| --- | --- | --- | --- | --- | --- |
| gi|4536667 | 4.77 | 53315.85 | COG0539J | rpsA | 30S ribosomal protein S1 [Mycobacterium smegmatis str. MC2 155] |
| gi|4536043 | 0.43 | 60718 | COG0100J | rpsK | 30S ribosomal protein S11 [Mycobacterium smegmatis str. MC2 155] |
| gi|4531431 | 10.78 | 14217.52 | COG0099J | rpsM | 30S ribosomal protein S13 [Mycobacterium smegmatis str. MC2 155] |
| gi|4534936 | 10.61 | 9516.1 | COG0238J | rpsR | 30S ribosomal protein S18 [Mycobacterium sp. MCS] |
| gi|4534380 | 10.48 | 10751 | COG0185J | [rpsS](http://www.ncbi.nlm.nih.gov/sites/entrez?db=gene&cmd=search&term=4534380&RID=MEU0WR0U012&log$=geneexplicitprot&blast_rank=1) | 30S ribosomal protein S19 [Mycobacterium smegmatis str. MC2 155] |
| gi|4535569 | 5.54 | 31761.07 | COG0052J | rpsB | 30S ribosomal protein S2 [Mycobacterium smegmatis str. MC2 155] |
| gi|4532601 | 10.2 | 30139.14 | COG0092J | rpsC | 30S ribosomal protein S3 [Mycobacterium smegmatis str. MC2 155] |
| gi|4535400 | 10.56 | 17600.37 | COG0049J | rpsG | 30S ribosomal protein S7 [Mycobacterium smegmatis str. MC2 155] |
| gi|4537206 | 6.86 | 29480 | COG1947I | ispE | 4-diphosphocytidyl-2C-methyl-D-erythritol kinase [Mycobacterium smegmatis str. MC2 155] |
| gi|4533313 | 9.64 | 24883 | COG0081J | rplA | 50S ribosomal protein L1 [Mycobacterium smegmatis str. MC2 155] |
| gi|4533916 | 10.23 | 13290.48 | COG0093J | rplN | 50S ribosomal protein L14 [Mycobacterium smegmatis str. MC2 155] |
| gi|4536702 | 10.52 | 15742.19 | COG0197J | rplP | 50S ribosomal protein L16 [Mycobacterium smegmatis str. MC2 155] |
| gi|4531572 | 11.39 | 30576.79 | COG0090J | rplB | 50S ribosomal protein L2 [Mycobacterium smegmatis str. MC2 155] |
| gi|4535328 | 10.95 | 16323.57 | COG0091J | MSMEG_1441 | 50S ribosomal protein L22 [Mycobacterium smegmatis str. MC2 155] |
| gi|4535014 | 4.55 | 13450 | COG0222J | rplL | 50S ribosomal protein L7/L12 [Mycobacterium smegmatis str. MC2 155] |
| gi|4535301 | 5.26 | 63099 | COG4770I | - | acetyl-/propionyl-coenzyme A carboxylase alpha chain [Mycobacterium smegmatis str. MC2 155] |
| gi|4537238 | 3.92 | 10578.8 | COG0236IQ | - | acyl carrier protein [Mycobacterium smegmatis str. MC2 155] |
| gi|4537593 | 4.51 | 35546 | - | - | alanine and proline-rich secreted protein apa [Mycobacterium smegmatis str. MC2 155] |
| gi|4532619 | 6.02 | 40314.37 | COG0003P | - | Anion-transporting ATPase [Mycobacterium smegmatis str. MC2 155] |
| gi|4532134 | 4.93 | 128267 | COG3320Q | - | NAD dependent epimerase/dehydratase family protein [Mycobacterium smegmatis str. MC2 155] |
| gi|4533381 | 5.67 | 31755 | COG0491R | - | beta-lactamase [Mycobacterium smegmatis str. MC2 155] |
| gi|4533718 | 4.31 | 19488.66 | COG0580G | - | Chain A, Main Porin From Mycobacteria Smegmatis (Mspa) |
| gi|4533338 | 4.81 | 56487.29 | COG0459O | groEL | chaperonin GroEL [Mycobacterium smegmatis str. MC2 155] |
| gi|4532222 | 4.51 | 10757 | COG0234O | groES | co-chaperonin GroES [Mycobacterium smegmatis str. MC2 155] |
| gi|4533253 | 4.91 | 7343 | COG1278K | - | cold-shock DNA-binding domain protein [Mycobacterium sp. MCS] |
| gi|4534426 | 4.9 | 54357.96 | COG0111HE | serA | D-3-phosphoglycerate dehydrogenase [Mycobacterium smegmatis str. MC2 155] |
| gi|4537128 | 4.65 | 61029.52 | COG0508C | sucB | dihydrolipoamide acetyltransferase [Mycobacterium smegmatis str. MC2 155] |
| gi|4532396 | 4.67 | 29544.39 | COG3599D | - | DivIVA protein [Mycobacterium smegmatis str. MC2 155] |
| gi|4534852 | 11.64 | 21217 | COG0776L | hup | DNA-binding protein HU [Mycobacterium smegmatis str. MC2 155] |
| gi|4532832 | 5.33 | 148152.5 | COG0085K | rpoC | DNA-directed RNA polymerase subunit beta' [Mycobacterium smegmatis str. MC2 155] |
| gi|4534885 | 4.77 | 31539 | COG2086C | etfA | electron transfer flavoprotein, alpha subunit [Mycobacterium smegmatis str. MC2 155] |
| gi|4534407 | 5.11 | 29388 | COG0264J | tsf | elongation factor Ts [Mycobacterium smegmatis str. MC2 155] |
| gi|4537531 | 5.18 | 43735.72 | COG0050J | tuf | elongation factor Tu [Mycobacterium smegmatis str. MC2 155] |
| gi|4536499 | 5.22 | 26054.85 | COG1024I | MSMEG_5639 | enoyl-CoA hydratase [Mycobacterium smegmatis str. MC2 155] |
| gi|4534918 | 4.53 | 60560.38 | COG0747E | MSMEG_0643 | extracellular solute-binding protein, family protein 5, putative [Mycobacterium smegmatis str. MC2 155] |
| gi|4534221 | 4.2 | 25659 | COG2335M | MSMEG_5196 | fasciclin domain protein [Mycobacterium smegmatis str. MC2 155] |
| gi|4532653 | 4.87 | 329334 | COG4981I | MSMEG_4757 | fatty acid synthase [Mycobacterium smegmatis str. MC2 155] |
| gi|4535975 | 5.32 | 18986 | COG0057G | gap | glyceraldehyde-3-phosphate dehydrogenase [Mycobacterium smegmatis] |
| gi|4535575 | 4.74 | 55098.4 | COG0554C | glpK | glycerol kinase [Mycobacterium smegmatis str. MC2 155] |
| gi|4533338 | 4.8 | 57566 | COG0459O | groEL | heat shock protein 65 [Mycobacterium smegmatis str. MC2 155] |
| gi|4532040 | 4.77 | 66640 | COG0443O | dnaK | heat shock protein Hsp70 [Mycobacterium smegmatis str. MC2 155] |
| gi|4535714 | 5.42 | 19169 | COG2186K | MSMEG_0874 | hypothetical protein Franean1_3631 [Mycobacterium smegmatis str. MC2 155] |
| gi|4535563 | 4.68 | 11663 | - | MSMEG_0243 | hypothetical protein MSMEG_0243 [Mycobacterium smegmatis str. MC2 155] |
| gi|4536050 | 4.98 | 15372 | - | MSMEG_2983 | hypothetical protein MSMEG_2983 [Mycobacterium smegmatis str. MC2 155] |
| gi|4530973 | 9.69 | 18140 | - | MSMEG_3766 | hypothetical protein MSMEG_3766 [Mycobacterium smegmatis str. MC2 155] |
| gi|4537170 | 4.85 | 35856 | COG0003P | MSMEG_6193 | hypothetical protein nfa3420 [Mycobacterium smegmatis str. MC2 155] |
| gi|4533720 | 4.86 | 36399 | COG0059EH | ilvC | ketol-acid reductoisomerase [Mycobacterium smegmatis str. MC2 155] |
| gi|4533551 | 5.47 | 37210 | COG1376S | MSMEG_0233 | lipoprotein Lpps [Mycobacterium smegmatis str. MC2 155] |
| gi|4537534 | 6.07 | 16408 | - | MSMEG_3903 | Low molecular weight antigen MTB12 [Mycobacterium smegmatis str. MC2 155] |
| gi|4535452 | 6.8 | 20102.71 | COG0377C | nuoB | NADH dehydrogenase subunit B [Mycobacterium smegmatis str. MC2 155] |
| gi|4531859 | 3.77 | 20343 | COG0652O | MSMEG_3434 | peptidyl-prolyl cis-trans isomerase, fkbp-type domain protein [Mycobacterium smegmatis str. MC2 155] |
| gi|4536396 | 4.49 | 44734 | COG0148G | eno | phosphopyruvate hydratase [Mycobacterium smegmatis str. MC2 155] |
| gi|4537728 | 4.69 | 39987 | COG0151F | purD | phosphoribosylamine--glycine ligase [Mycobacterium smegmatis str. MC2 155] |
| gi|4531096 | 4.6 | 194466.9 | COG3319Q, COG3321Q | MSMEG_6392 | polyketide synthase [Mycobacterium smegmatis str. MC2 155] |
| gi|4532100 | 5.46 | 60286 | COG0318IQ | MSMEG_5291 | probable acyl-CoA synthetases [Mycobacterium smegmatis str. MC2 155] |
| gi|4536033 | 4.98 | 54468 | COG1597IR | glpK | putative glycerol kinase [Mycobacterium smegmatis str. MC2 155] |
| gi|4531591 | 10.15 | 14353 | - | MSMEG_1060 | putative Lsr2 protein [Mycobacterium smegmatis str. MC2 155] |
| gi|4533916 | 10.36 | 13289 | COG0093J | rplN | ribosomal protein L14 [Mycobacterium sp. MCS] |
| gi|4534083 | 5.03 | 54010 | COG1028IQR | MSMEG_6026 | S-adenosyl-L-homocysteine hydrolase [Mycobacterium smegmatis str. MC2 155] |
| gi|4534426 | 4.86 | 54475 | COG0111 | serA | SerA [Mycobacterium smegmatis str. MC2 155] |
| gi|4535810 | 4.7 | 40907 | COG0045C | sucC | succinyl-CoA synthetase subunit beta [Mycobacterium smegmatis str. MC2 155] |
| gi|4531491 | 6.71 | 71747.63 | COG1158K | rho | transcription termination factor Rho [Mycobacterium smegmatis str. MC2 155] |
| gi|4536439 | 4.3 | 51655.34 | COG0544O | tig | trigger factor [Mycobacterium smegmatis str. MC2 155] |
